# Dll4 assembles the umbilical cord and placental vasculature

**DOI:** 10.1172/jci.insight.194461

**Published:** 2026-02-17

**Authors:** Derek C. Sung, Hana A. Ahanger, Sweta Narayan, Jesse A. Pace, Mei Chen, Jisheng Yang, Siqi Gao, T.C.S. Keller, Jenna Bockman, Xiaowen Chen, Erica Nguyen, Alan T. Tang, Patricia Mericko-Ishizuka, Ivan Maillard, Mark L. Kahn

**Affiliations:** 1 Cardiovascular Institute, Department of Medicine, and; 2 Department of Pathology and Laboratory Medicine, Perelman School of Medicine, University of Pennsylvania, Philadelphia, Pennsylvania, USA.; 3 Division of Hematologic Malignancies, Department of Medicine & Human Oncology and Pathogenesis Program, Memorial Sloan Kettering Cancer Center, New York, New York, USA.

**Keywords:** Development, Reproductive biology, Vascular biology, Angiogenesis, Endothelial cells

## Abstract

Proper development of the umbilical cord and placental vasculature is essential for embryonic development. While the allantois is known give rise to endothelial cells (ECs) within the placenta, whether the allantois gives rise to ECs in the umbilical cord is debated. Furthermore, a lack of genetic tools to study placental vascular development independent of the embryo proper has hindered robust investigation into the primary cause of vascular defects from early studies utilizing global KOs. In this study, we delineate the contribution of the allantois to the umbilical vessels and utilize a mouse genetic tool previously developed by our lab to revisit the role of Notch signaling during placental development. We show that the allantois has mosaic contribution to the umbilical endothelium with higher contributions closer to the placenta. Allantoic deletion of *Dll4* disrupts umbilical cord and placental vascular formation with secondary defects in the heart. Lastly, we identify *Unc5b* downstream of Notch signaling that restricts EC migration while promoting chemokine signaling for vascular smooth muscle cell (vSMC) recruitment to arteries. These findings identify a genetic tool for investigating placental vascular development and give insights into the ontogeny and mechanisms of placental vascular and umbilical cord development.

## Abstract 2

Proper formation of the placenta and umbilical cord are essential for fetal growth. Notch-chemokine signaling plays an essential role in building the placental vasculature.

## Introduction

The placenta is a vascular organ of pregnancy that plays a central role in embryonic growth and development by mediating nutrient, gas, and waste exchange at the maternal-fetal interface. Placental dysfunction contributes to pregnancy-related disorders and pathologies such as preeclampsia, intrauterine growth restriction, developmental defects, and fetal demise. However, the underlying mechanisms of these diseases are poorly understood. Establishment of the maternal-fetal interface in humans and mice requires coordination between multiple cell types to ensure the formation of 2 distinct yet deeply intertwined vascular systems: trophoblast-lined sinuses carrying maternal blood and fetal endothelial-lined capillaries carrying fetal blood (1). Furthermore, these trophoblast and endothelial-lined networks must also connect to their respective blood supplies via spiral arteries and the umbilical cord, respectively. Failure to establish these vasculatures or their connections can result in fetal growth restriction and death (2).

The umbilical cord and fetal placental vasculature originate from the allantois, a mesoderm-derived structure born from the primitive streak. At around E8.5, the allantois merges with the chorion through a process appropriately termed chorioallantoic fusion, which subsequently gives rise to the placental and umbilical vessels (1, 3, 4). In the murine placenta, these vessels carrying fetal blood are found in the labyrinth layer where they intermingle with trophoblasts carrying maternal blood (1, 3, 5). Rapid establishment of the umbilical cord and expansion of the placental vasculature following chorioallantoic fusion is critical as the main source of nutrition shifts from the yolk sac to the placenta at around E9.5 (1). However, the mechanisms by which the allantois gives rise to the vessels in the umbilical cord and placenta are not well understood. Furthermore, given that the umbilical cord bridges the placenta and embryo, it remains unknown how much of the umbilical cord is derived from the allantois versus embryo proper.

The Notch signaling pathway is a key regulator of vascular development whereby activation of Notch receptors by the membrane-bound delta-like ligand 4 (DLL4) controls arterial identity and sprouting angiogenesis (6–9). DLL4 is expressed in arterial endothelial cells (ECs) and tip cells (10–13). During development, *Dll4* haploinsufficiency in mice causes aortic atresia and arterial regression that extends to the venous circulation, ultimately resulting in pericardial edema and embryonic demise by E10.5 (14–16). In the retina, deletion of *Dll4* induces hypersprouting of the capillaries in the angiogenic front by upregulation of VEGF receptors (10, 11, 17, 18). DLL4 binds and activates NOTCH1 to promote EC quiescence, lumen formation, and arterial specification (19, 20), and deletion of *Notch1* results in embryonic lethality between E9.5 and E10.5 with major placental and cardiovascular defects reminiscent of loss of *Dll4* (6, 7, 16, 21).

Many early embryonic lethal phenotypes, particularly cardiovascular defects, are associated with and can be caused by placental abnormalities (22). Notably, much of the phenotypic analysis into DLL4/Notch signaling in vascular development in the embryo proper was conducted using global KOs or constitutive endothelial Cre lines that are also active in the yolk sac and placental vasculature (6). However, the role of DLL4/Notch in formation of the placental vasculature has never been closely examined, and lack of genetic tools have precluded robust investigation into how the placental vasculature develops independent of the embryo proper. Our group recently developed a *Hoxa13^Cre^* mouse that specifically targets the allantois and its progeny in the placenta and umbilical cord (23). We therefore sought to determine the role of *Dll4* in placental vascular growth after chorioallantoic fusion and whether the early embryonic lethality examined in *Dll4*-KO mice could be at least partially explained by placental defects.

In this study, we demonstrate that the allantois gives rise to both placental and umbilical cord ECs and vascular smooth muscle cells (vSMCs). Targeted deletion of *Dll4* in the placental vasculature and umbilical cord using the *Hoxa13^Cre^* mouse disrupted umbilical and placental artery formation, resulting in secondary cardiovascular failure and early embryonic death. RNA-seq identified *Unc5b* and chemokine signaling downstream of *Dll4*. Lastly, in vivo disruption of endothelial chemokine signaling reduced vSMC recruitment to arteries. Altogether, we find that DLL4 couples formation of the umbilical vessels with the growing placental fetal vascular plexus in part through *Unc5b*-chemokine signaling.

## Results

### *Defining the contributions of the allantois to placental and umbilical vessels*.

**Figure 1:**
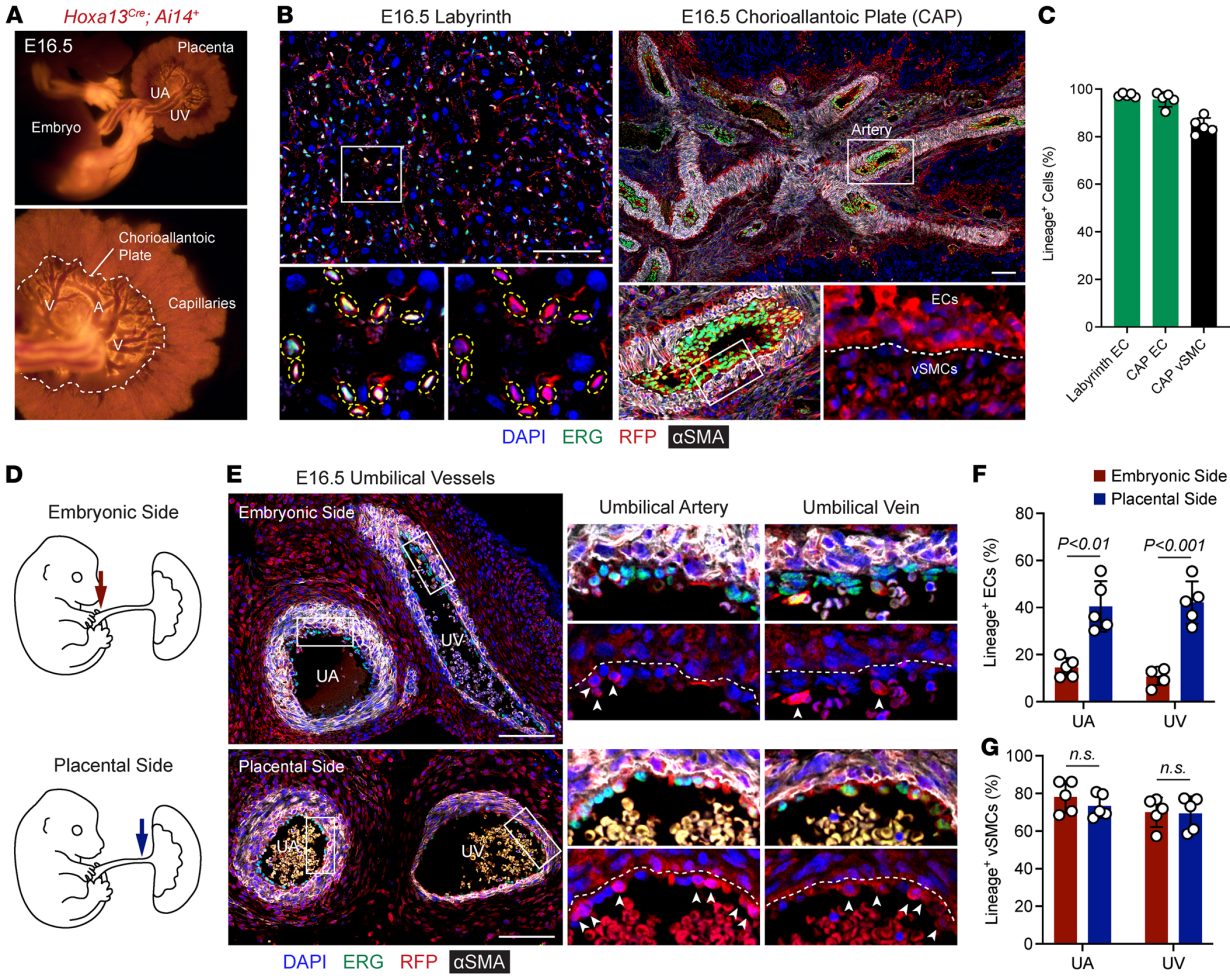
The allantois gives rise to endothelial cells in the umbilical cord and placenta. (**A**) Wholemount imaging of E16.5 *Hoxa13^Cre^;Ai14* embryo and placenta. (**B**) Immunofluorescence staining of E16.5 labyrinth capillaries and chorioallantoic plate (CAP) vessels for ERG (green), lineage/RFP (red), and αSMA (gray). Yellow dotted circles indicate lineage-positive (RFP^+^) ECs in the labyrinth. White dashed line demarcates endothelial cells (ECs) and vascular smooth muscle cells (vSMCs). Scale bars: 100 μm. (**C**) Quantification of lineage-positive (RFP^+^) ECs and vSMCs in the chorioallantoic plate (*n* = 5 placentas). (**D**) Schematic of regions of the umbilical cord near the embryo (red arrow) and placenta (blue arrow) subjected to immunofluorescence staining. (**E**) Immunofluorescence staining of E16.5 umbilical vessels for ERG (green), lineage/RFP (red), and αSMA (gray). Dotted line demarcates EC from vSMCs. UA, umbilical artery; UV, umbilical vein. Scale bars: 100 μm. (**F** and **G**) Quantification of lineage-positive (RFP^+^) ECs (**F**) and vSMCs (**G**) in the umbilical vessels in regions close to the embryo and placenta (*n* = 5 placentas). Data represent mean ± SD. An unpaired *t* test was performed for statistical analysis.

The fetal vascular plexus in the placenta and umbilical cord are primarily derived from the allantois (1, 3, 4). The specific cell types derived from the allantois have not been well defined, and the exact contributions of the allantois to the umbilical cord is debated (24–26). To better characterize the cell types the allantois gives rise to in the umbilical cord and placental vasculature, we utilized the *Hoxa13^Cre^* mouse, a tool previously developed by our lab that is active specifically in the allantois (23). Since the placental vasculature is established by late mouse gestation, *Hoxa13^Cre^* mice were crossed with an *Ai14* red fluorescent protein (RFP) reporter and placentas and embryos examined at E16.5. RFP positivity was found within the entirety of the umbilical vessels, the major vessels of the chorioallantoic plate (CAP) that branch from the umbilical vessels, and the capillary tufts at E16.5 (Figure 1A). Additional RFP-positive signal was noted in the limbs and tail, although immunofluorescence staining showed this was due to bone and mesenchymal cells and not ECs (Figure 1A and Supplemental Figure 1; supplemental material available online with this article; https://doi.org/10.1172/jci.insight.194461DS1).

Immunostaining for RFP (lineage-positive cells), ERG (EC nuclei), and αSMA (for vSMCs) on umbilical cord and placenta sections demonstrated placental capillary ECs, and the ECs and vSMCs of major CAP vessels were almost entirely lineage positive (97.5%, 95.7%, and 84.5%, respectively; Figure 1, B and C), suggesting that these cells are mostly derived from *Hoxa13^+^* progenitors in the allantois. The CAP vessels connect to the umbilical cord at the base of the placenta. In the umbilical cord, there were differences in RFP-positive ECs based on proximity to the placenta versus embryo (Figure 1D) with more lineage-positive ECs in the umbilical cord on the placental side (40.4% for umbilical artery and 42.1% for umbilical vein) compared with the embryonic side (14.5% for UA, 10.7% for UV) (Figure 1, E and F). There were no differences between the umbilical artery and vein at either location, or in lineage-positive vSMCs between umbilical artery or vessel at either location (Figure 1, E–G). These studies demonstrate differential contributions of the allantois to vessels in the placenta and umbilical cord, with almost all ECs in the placenta being derived from the allantois while only a portion of ECs in the umbilical cord are from these cells in the allantois.

### *Allantois-specific deletion of Dll4 causes cardiovascular defects and embryonic lethality by E11.5*.

**Figure 2:**
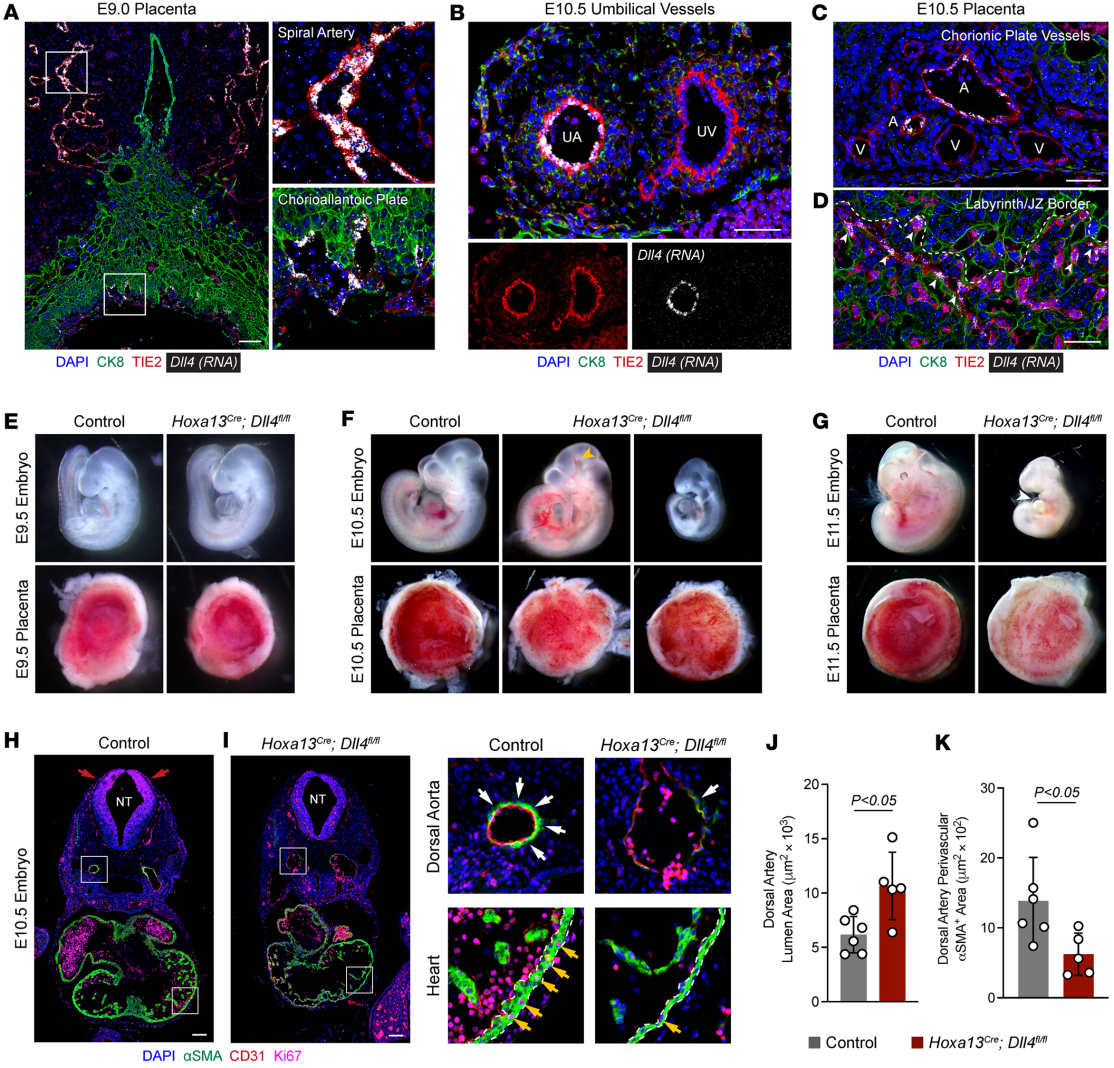
*Dll4* is expressed in umbilical and placental arteries and required for embryonic survival. (**A**) Immunofluorescence staining and RNA-FISH of E9.0 placenta for CK8 (green), TIE2 (red), and *Dll4* (gray, RNA). Scale bars: 100 μm. (**B**–**D**) Immunofluorescence staining and RNA-FISH of E10.5 umbilical cord (**B**) and placenta (**C** and **D**) for CK8 (green), TIE2 (red), and *Dll4* (gray, RNA). White arrowheads point to *Dll4^+^* endothelial cells near the labyrinth-junctional zone border (dashed line). UA, umbilical artery; UV, umbilical vein; A, artery; V, vein. Scale bars: 50 μm. (**E**–**G**) Gross images of control and *Hoxa13^Cre^;Dll4^fl/fl^* embryos and placentas at E9.5 (**E**), E10.5 (**F**), and E11.5 (**G**). Yellow arrowhead points to vascular congestion. White arrowhead points to pericardial edema. (**H** and **I**) Immunofluorescence staining of E10.5 control and *Hoxa13^Cre^;Dll4^fl/fl^* embryos for αSMA (green), CD31 (red), and Ki67 (magenta). Red arrows point to Ki67^+^ regions in the neural tube (NT). White arrows point to αSMA^+^ surrounding the dorsal aorta. Yellow arrows point to Ki67^+^ cardiomyocytes. Scale bars: 100 μm. (**J** and **K**) Quantification of dorsal aorta lumen area and perivascular αSMA^+^ smooth muscle cells (*n* = 5–6 embryos per genotype). Data represent mean ± SD. An unpaired *t* test was performed for statistical analysis.

Notch signaling is a well-appreciated signaling pathway that regulates vascular development (6–9). While the Notch-activating ligand DLL4 has been extensively investigated in the embryonic and postnatal vasculature and is known to be highly expressed in arteries and tip cells (7, 10–13, 15, 16), its expression and function in the placenta are much less defined. RNA fluorescent in situ hybridization (FISH) costained with TIE2 to mark ECs and Cytokeratin 8 (CK8) to mark trophoblasts delineated the temporal and spatial expression patterns of *Dll4* in the placenta. At E9.0, *Dll4* mRNA expression was restricted to maternal spiral arteries and some ECs of the primitive vascular plexus in the CAP (Figure 2A). By E10.5, *Dll4* expression was highest in ECs of the umbilical artery and the placental arteries within the CAP (Figure 2, B and C). The capillaries close to the border of the junctional zone also expressed *Dll4*, albeit to a lesser degree (Figure 2D). These data are consistent with analysis of single-cell RNA-seq data from Liang et al. (27), demonstrating highest *Dll4* expression in arteries followed by capillaries and absent expression in veins (Supplemental Figure 2). In contrast, examination of various Notch receptors in placental single-nucleus RNA-seq (28) shows variable expression amongst cell types, with the main DLL4 binding partner *Notch1* expressed in ECs and vSMCs (Supplemental Figure 3). In summary, expression of *Dll4* in the placenta largely mirrors its expression in the embryonic and postnatal vasculature.

Establishment of the placental vasculature and umbilical vessels occurs rapidly following chorioallantoic fusion to permit the onset of maternal-fetal nutrient exchange. *Hoxa13^Cre^* and *Dll4* floxed mice were crossed to generate *Hoxa13^^Cre^^;Dll4^fl/fl^* (*Dll4*-knockout) mice where *Dll4* is deleted specifically in the allantois-derived vasculature. Mendelian ratio analysis at E9.5, E10.5, and E11.5 revealed presence of all genotypes at expected percentages with nonsignificant *P* values (*P* > 0.05) by Fisher’s exact test (Table 1). However, no *Hoxa13^^Cre^^;Dll4^fl/fl^* embryos were found from E12.5–E17.5 or at P21 (Table 1) indicating that they died and were resorbed by E12.5. At E9.5, control and *Hoxa13^^Cre^^;Dll4^fl/fl^* embryos and placentas were indistinguishable (Figure 2E). However, by E10.5, *Dll4*-KO placentas were paler, and all embryos were viable with beating hearts but exhibited variable phenotypes, with some demonstrating embryonic vascular congestion (8 of 11) and some with severe fetal growth restriction (3 of 11) (Figure 2F). At E11.5, all embryos (9 of 9) were dead with markedly pale placentas and pericardial edema (white arrowhead, Figure 2G). The variable embryonic phenotypes beginning at E10.5 coupled with consistently pale placentas suggested a primary placenta defect contributing to embryonic demise.

Immunostaining for αSMA (vSMCs and myocardium), CD31 (ECs), and Ki67 (proliferating cells) in control and mutant embryos at E10.5 (excluding severely growth restricted embryos) showed numerous defects. First, the neural tube had markedly fewer Ki67^^+^^ cells demonstrating decreased proliferation (Figure 2, H and I). Second, dorsal aortas in *Hoxa13^^Cre^^;Dll4^fl/fl^* embryos were present but dilated with reduced vSMC coverage (Figure 2, H–K). Interestingly, this is in contrast to global *Dll4* KOs with collapsed dorsal aortas (29). While *Dll4* has been shown to have gene dosage effects (15), comparison of dorsal aortas in Cre^^–^^ (*Dll4^fl/+^* and *Dll4^fl/fl^*) and Cre^^+^^ heterozygous (*Hoxa13^^Cre^^;Dll4^fl/+^*) embryos showed no differences (Supplemental Figure 4A). Lastly, *Hoxa13^^Cre^^;Dll4^fl/fl^* embryos had ventricular thinning with reduced myocardial proliferation (Figure 2, H and I), similar to global *Dll4* KOs. These findings are consistent with global growth restriction secondary to placental-specific loss of *Dll4* and suggest that while arterial collapse in global *Dll4* mutants is a primary defect, reported heart phenotypes are likely secondary to placental defects.

### *Dll4 is required for umbilical cord development*.

**Figure 3:**
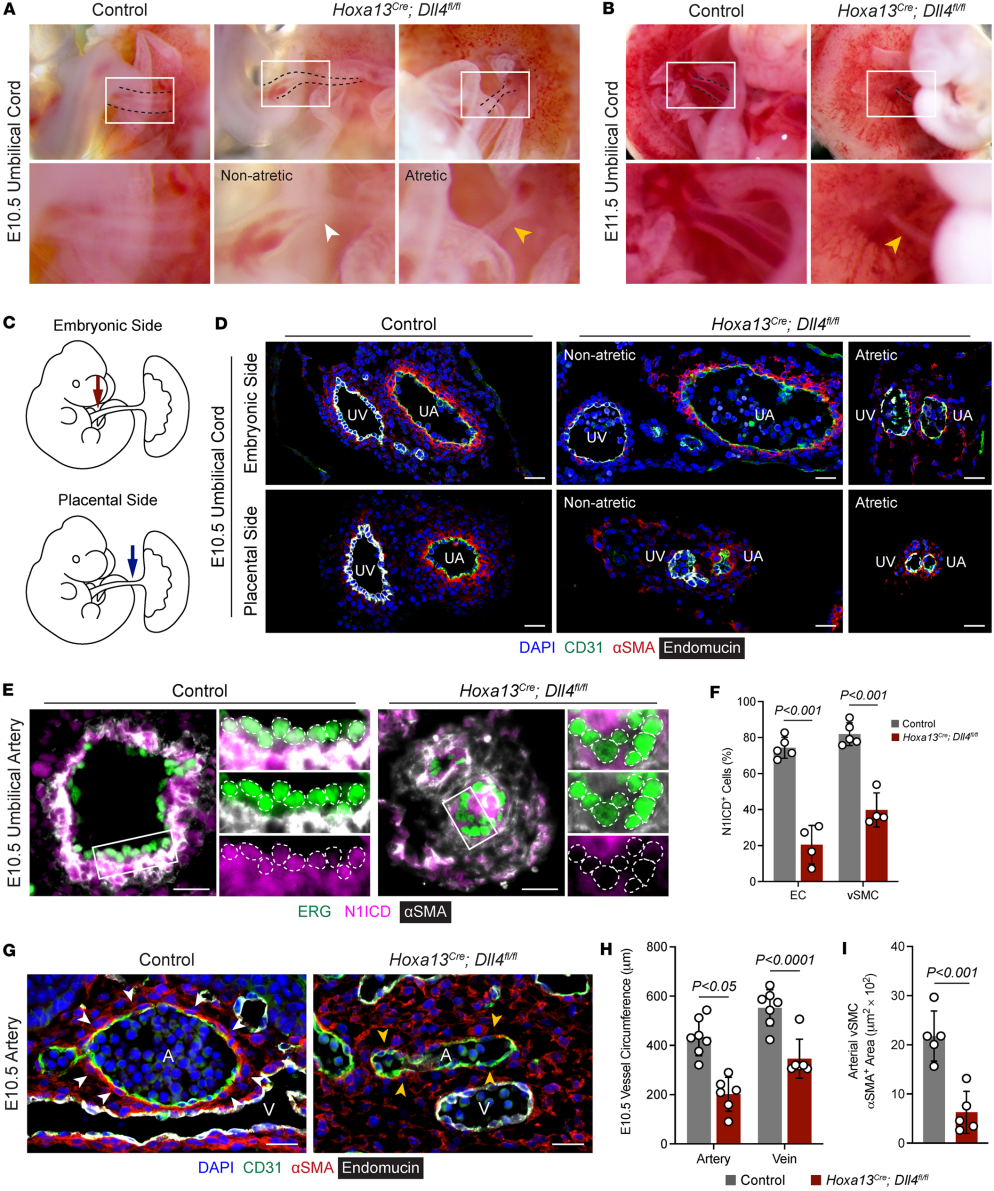
Deletion of *Dll4* causes umbilical cord atresia and disrupts placental vascular organization. (**A** and **B**) Gross images of control and *Hoxa13^Cre^;Dll4^fl/fl^* umbilical cords connecting embryos and placentas at E10.5 (**A**) and E11.5 (**B**). At E10.5, most (8 of 11) *Hoxa13^Cre^;Dll4^fl/fl^* cords narrowed with restricted blood flow (white arrowhead) as they inserted into the placenta while the rest (3 of 11) were completely atretic (yellow arrowhead). At E11.5, all *Hoxa13^Cre^;Dll4^fl/fl^* cords had undergone atresia (yellow arrowhead). (**C**) Schematic of regions of the umbilical cord near the embryo (red arrow) and placenta (blue arrow) subjected to immunofluorescence staining. (**D**) Immunofluorescence staining of E10.5 control and *Hoxa13^Cre^;Dll4^fl/fl^* (atretic and nonatretic) umbilical cords for CD31 (green), αSMA (red), and Endomucin (gray). UA, umbilical artery; UV, umbilical vein. Scale bars: 25 μm. (**E** and **F**) Immunofluorescence staining of umbilical arteries from E10.5 control and *Hoxa13^Cre^;Dll4^fl/fl^* placentas for αSMA (gray), ERG (green), and NOTCH1-intracellular domain (N1ICD, magenta) and quantification of N1ICD^+^ ECs and vSMCs (*n* = 4–5 placentas per genotype). Scale bars: 25 μm. (**G**) Immunofluorescence staining of vessels in the chorioallantoic plate in E10.5 control and *Hoxa13^Cre^;Dll4^fl/fl^* placentas for CD31 (green), αSMA (red), and Endomucin (gray). White arrowheads point to abundant αSMA^+^ vSMCs surrounding control arteries whereas yellow arrowheads point to decreased vSMCs in *Hoxa13^Cre^;Dll4^fl/fl^* arteries. A, artery; V, vein. Scale bars: 25 μm. (**H**) Quantification of E10.5 arterial and venous vessel circumference (*n* = 5–7 placentas per genotype, each data point represents the average of at least 3 vessels per placenta). (**I**) Quantification of perivascular arterial αSMA^+^ smooth muscle cells (*n* = 5 placentas per genotype, each data point represents the average of at least 3 vessels per placenta). Data represent mean ± SD. An unpaired *t* test was performed for statistical analysis.

The studies above demonstrate that the allantois gives rise to ECs in the umbilical cord, CAP, and placental capillaries, and the umbilical and CAP arteries highly express *Dll4* while only a subset of placental capillaries express a moderate level of *Dll4*. During dissections of embryos and placentas, we noticed that the umbilical cords of *Dll4*-KO placentas were grossly abnormal. E10.5 embryos that had vascular congestion but were not growth restricted had umbilical cords that narrowed as they entered the placenta with pinched off blood flow (Figure 3A). In contrast, growth restricted E10.5 embryos had umbilical cords that appeared atretic and were devoid of any blood (Figure 3A). Umbilical cords of all mutant embryos were completely atretic by E11.5 (Figure 3B).

Immunostaining of the umbilical cord for Endomucin, CD31, and αSMA enabled differentiation of arteries (CD31^^+^^, Endomucin^^lo^^ ECs surrounded by many αSMA^^+^^ vSMCs) from veins (CD31^^+^^, Endomucin^^hi^^ ECs surrounded by few αSMA^^+^^ vSMCs). We specifically looked at the umbilical cord at 2 areas: the embryonic side and placental side (Figure 3C). *Dll4* KOs with nonatretic umbilical cords had relatively normal-appearing but mildly dilated arteries on the embryonic side (Figure 3D). In contrast on the placental side, arteries and veins were markedly diminished with small, nearly collapsed lumens lacking vSMC coverage. *Dll4* KOs with atretic umbilical cords had diminished vessels on both the embryonic and placental sides (Figure 3D). Lastly, NOTCH1-intracellular domain (N1ICD) was decreased in ECs and vSMCs of KO umbilical arteries (Figure 3, E and F), demonstrating that *Dll4* deletion in ECs reduced Notch signaling in ECs and vSMCs. These studies reveal that embryonic growth restriction occurs after umbilical artery atresia and that umbilical vessel degeneration starts closer to the placenta and extends toward the embryo.

### *Dll4 controls vascular organization in the CAP and labyrinth*.

The umbilical cord inserts into the placenta at the CAP where it branches into the major placental arteries and veins. The studies above demonstrate that loss of *Dll4* disrupts umbilical cord development where almost half of the ECs are derived from the allantois. We therefore wondered how loss of *Dll4* might affect the placental vasculature in the CAP, where nearly all the ECs originate from the allantois. Immunofluorescence studies demonstrated that *Dll4* KOs exhibited a reduction in vessel circumference in both arteries and veins, with a greater decrease in arteries relative to veins (54% versus 37% reduction; Figure 3, G and H). Interestingly, heterozygous controls had a slight decrease in arterial but not venous circumference compared with Cre^^–^^ controls (Supplemental Figure 4B), suggesting a slight gene dosage effect. Furthermore, arteries had fewer associated αSMA^^+^^ vSMCs (Figure 3, G and I, and Supplemental Figure 4D). These data demonstrate that *Dll4* is required for proper formation of large vessels that branch from the umbilical cord following insertion into the placenta.

**Figure 4:**
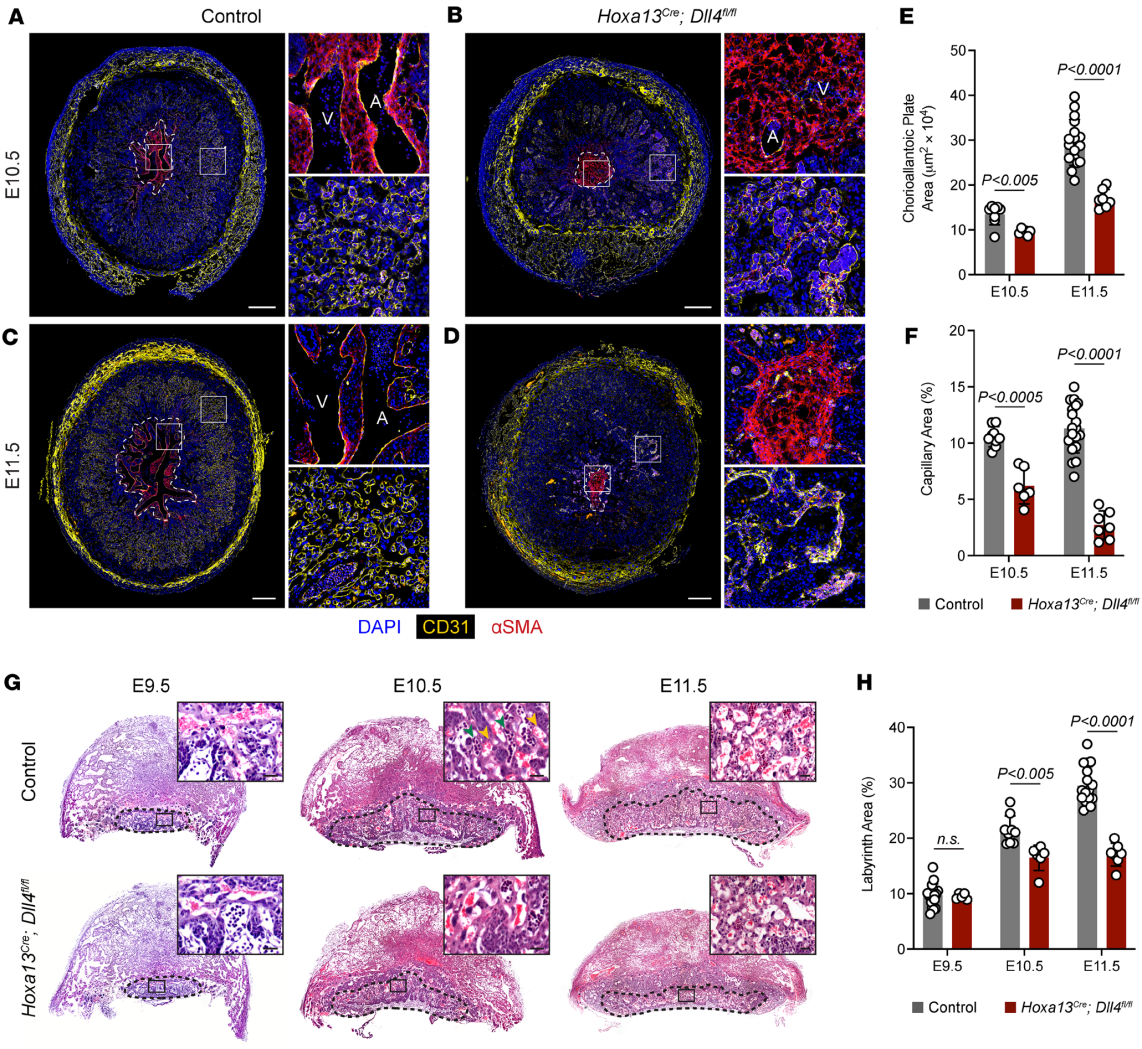
*Dll4* is required for expansion of the placental labyrinth and syncytiotrophoblast patterning. (**A**–**D**) Immunofluorescence staining of E10.5 and E11.5 control and *Hoxa13^Cre^;Dll4^fl/fl^* en face placental sections for CD31 (yellow) and αSMA (red). Boxes show magnified regions of the chorioalantoic plate. Dotted line outlines the chorioallantoic plate. Yellow arrowheads point to dilated (E10.5) capillaries that become collapsed (E11.5). A, artery; V, vein. Scale bars: 500 μm. (**E** and **F**) Quantification of the chorioallantoic plate area (**E**) and capillary area in the labyrinth (**F**) (*n* = 6–19 placentas per genotype). (**G**) H&E staining of E9.5, E10.5, and E11.5 control and *Hoxa13^Cre^;Dll4^fl/fl^* placentas. Dotted lines demarcate the labyrinth region of the placenta. Scale bars: 50 μm. (**H**) Quantification of labyrinth area as a percentage of total cross-sectional area (*n* = 5–17 placentas per genotype). Data represent mean ± SD. An unpaired *t* test was performed for statistical analysis.

Vascular branching patterns, visualized by αSMA staining, in the CAP of *Dll4*-KO placentas were also affected and much smaller compared with controls at E10.5 and E11.5 (Figure 4, A–E). Control placentas at E10.5 and E11.5 had clearly identifiable arteries and veins that branched into smaller arterioles and venules in an organized manner, and then finally into capillaries (Figure 4, A–D). At E10.5, *Dll4* KOs had a few identifiable arteries and veins within the CAP but had few or no branches while distal capillaries were dilated and filled with blood (Figure 4B). By E11.5, the CAP vessels had completely degenerated and the distal capillaries had collapsed (Figure 4D). Heterozygous controls had no differences in CAP area but had a mild decrease in capillary area at E11.5 compared with Cre^^–^^ controls (Supplemental Figure 4, C and E).

Lastly, we examined the placental labyrinth and capillaries where maternal-fetal nutrient exchange takes place. Immunostaining for capillaries with Endomucin demonstrated a 41% decrease in capillary area in *Dll4* KOs relative to controls at E10.5 and a 76% decrease at E11.5 (Figure 4, A–D and F). Quantification of labyrinth area on H&E-stained sections showed no differences in labyrinth size by percent area at E9.5, but *Dll4*-KO placentas had decreased labyrinth size at E10.5 and E11.5 (Figure 4, G and H). No differences were found between heterozygous and Cre^^–^^ controls (Supplemental Figure 4F). Comparison of heterozygous and Cre^^–^^ controls overall demonstrates a slight gene-dosage effect that affects arteries and capillaries but not overall CAP area, labyrinth area, or embryo development. Altogether, these studies demarcate a specific pattern of vascular degeneration whereby the major vessels of the umbilical cord and CAP fail to recruit vSMCs and subsequently degenerate. This then causes vascular congestion in both the embryo and placenta, leading to nutrient deprivation, vascular collapse, and ultimately fetal demise.

### *UNC5B is downstream of DLL4 to restrict endothelial migration*.

**Figure 5:**
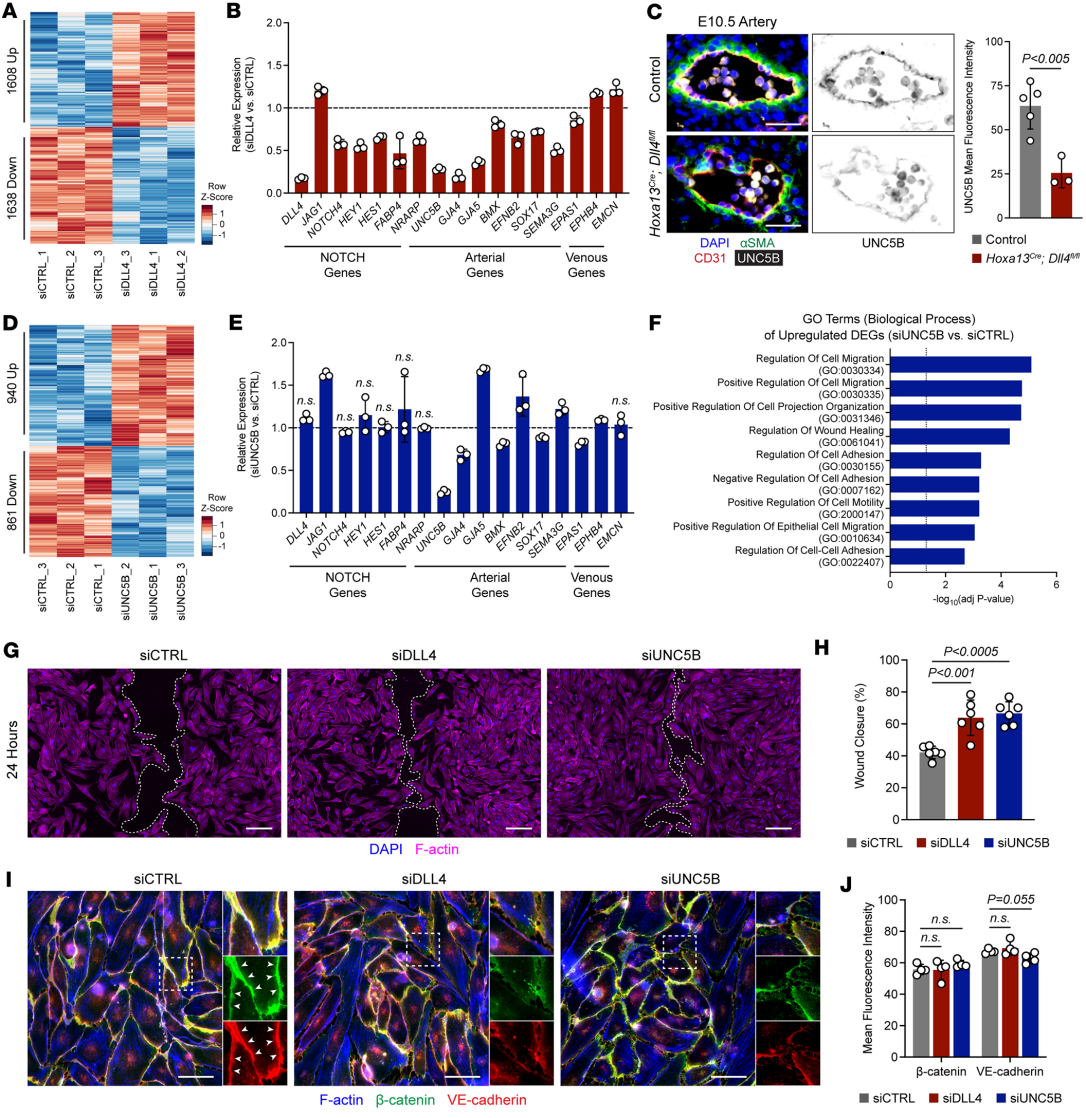
UNC5B acts downstream of DLL4 to restrict endothelial migration by stabilizing cell-cell junctions. (**A**) Heatmap of significantly (*P*_adj_ < 0.05) upregulated and downregulated genes comparing HUAECs treated with siDLL4 relative to siCTRL (*n* = 3 independent experiments per condition). (**B**) Expression of specific NOTCH, arterial, and venous genes in siDLL4-treated HUAECs relative to siCTRL. All genes are significant with an adjusted *P* < 0.05. (**C**) Immunofluorescence staining of arteries from E10.5 control and *Hoxa13^Cre^;Dll4^fl/fl^* placentas for αSMA (green), CD31 (red), and UNC5B (gray) and quantification of mean fluorescence intensity (*n* = 3–5 placentas per genotype, each data point represents the average of at least 3 vessels per placenta). Scale bars: 25 μm. (**D**) Heatmap of significantly (*P*_adj_ < 0.05) upregulated and downregulated genes comparing HUAECs treated with siUNC5B relative to siCTRL (*n* = 3 independent experiments per condition). (**E**) Expression of specific NOTCH, arterial, and venous genes in siUNC5B-treated HUAECs relative to siCTRL. All genes are significant with an adjusted *P* < 0.05 unless denoted with n.s. (not significant). (**F**) Gene ontology (GO) analysis (biological process) of the 940 upregulated genes in siUNC5B-treated HUAECs related to cell migration and cell adhesion. Dotted line indicates the threshold for significance. (**G**) Phalloidin (F-actin, magenta) stained HUAECs treated with siCTRL, siDLL4, or siUNC5B following 24 hours of wound closure (*n* = 6 independent experiments per condition). Dotted lines demarcate leading edge. Scale bars: 200 μm. (**H**) Quantification of percent wound closure in siCTRL, siDLL4, and siUNC5B HUAECs after 24 hours. (**I**) Immunofluorescence staining of HUAECs treated with siCTRL, siDLL4, or siUNC5B for β-catenin (green), VE-cadherin (red), and F-actin (blue). White arrowheads point to continuous cell-cell junctions in siCTRL cells that are more frequently disrupted in siDLL4 and siUNC5B cells. Scale bars: 50 μm. (**J**) Quantification of mean fluorescence intensity of β-catenin and VE-cadherin (*n*= 4 independent experiments per condition). Data represent mean ± SD. A 1-way ANOVA with post hoc Tukey HSD was performed for statistical analysis.

To further interrogate possible mechanisms downstream of *Dll4*, we utilized human umbilical arterial ECs (HUAECs) and performed bulk RNA-seq following 48-hour knockdown with an siRNA against *DLL4* (siDLL4) with adequate principal component analysis (PCA) clustering (Supplemental Figure 5A). *DLL4* knockdown resulted in significant (*P*__adj__ < 0.05) upregulation of 1,608 genes and downregulation of 1,638 genes (Figure 5A). As expected, *DLL4* was among the top significantly downregulated genes. Multiple other NOTCH genes and targets were downregulated (*NOTCH4*, *HEY1*, *HES1*, *FABP4*, *NRARP*) while the NOTCH ligand *JAG1* was upregulated (Figure 5B). Expression of multiple arterial genes (*UNC5B*, *GJA4*, *GJA5*, *BMX*, *EFNB2*, *SOX17*, *SEMA3G*, *EPAS1*) were downregulated and some venous genes (*EPHB4*, *EMCN*) (Figure 5B) were upregulated. Consistent with other studies, this suggests that *DLL4* is responsible for controlling arterial identity.

In particular, we were interested in *UNC5B*, part of the netrin family of receptors that had a greater than 3.5-fold decrease in gene expression in response to *DLL4* knockdown (Figure 5B). In the mouse retina, UNC5B has similar expression patterns to DLL4, where it is expressed in tip cells and arteries and functions in promoting the blood-retina barrier (30). Similarly, expression patterns of *Unc5b* in placenta ECs closely mirrored *Dll4* with predominant expression in arteries followed by capillaries (Supplemental Figure 5B). In mice, global or endothelial-specific deletion of *Unc5b* causes embryonic lethality around E12.5 (31–33). Interestingly, the apparent cause of embryonic lethality is due to defects in placental arteriogenesis rather than vascular defects in the embryo proper (31, 32). However, whether *Dll4*-mediated Notch signals modulate *Unc5b* is unclear. Given the similarities in expression patterns and phenotypes between *Dll4-* and *Unc5b*-KO mice and that knockdown of *DLL4* in HUAECs resulted in decreased *UNC5B*, *Dll4* may be signaling through *Unc5b* to contribute to vascular development.

Immunofluorescence staining for UNC5B showed that, compared with controls, there was a 2.5-fold decrease in arterial UNC5B expression in *Dll4*-KO placentas (Figure 5C). Bulk RNA-seq on siUNC5B-treated HUAECs with adequate PCA clustering (Supplemental Figure 5A) demonstrated 940 upregulated and 861 downregulated genes (*P*__adj__ < 0.05; Figure 5D). There was a greater than 4-fold decrease in *UNC5B* but no changes in most NOTCH target genes, including *DLL4* (Figure 5E). Some arterial genes were downregulated (*GJA4*, *BMX*, *SOX17*, *EPAS1*), although to a lesser degree, while other arterial genes were upregulated (*GJA5*, *EFNB2*, *SEMA3G*) and *EPHB4* was slightly increased (Figure 5E). Double knockdown of siDLL4/UNC5B more closely mirrored arterial gene expression patterns of siDLL4 alone (Supplemental Figure 5C), suggesting that *DLL4* controls global arterial identity while *UNC5B* affects only a subset of arterial genes.

Gene ontology (GO) analysis of upregulated genes in siUNC5B-treated HUAECs found multiple biological process terms related to promoting cell migration and regulation of cell adhesion (Figure 5F and Supplemental Table 1). Similarly, GO analysis of upregulated genes in siDLL4-treated HUAECs yielded similar cell migration and adhesion-related terms (Supplemental Figure 5D and Supplemental Table 2). In vitro wound closure studies using cell inserts and siRNA-mediated knockdown in HUAECs showed that knockdown of either *DLL4* or *UNC5B* increased rates of wound closure over a 24-hour period (Figure 5, G and H). Consistent with GO analysis, siDLL4- and siUNC5B-treated ECs had decreased junctional β-catenin and VE-cadherin relative to siCTRL ECs but no or slight decrease in overall protein levels (Figure 5, I and J). These data indicate that *DLL4* and *UNC5B* function in inhibiting cell migration by stabilizing cell-cell junctions.

### *UNC5B controls endothelial chemokine signaling to promote vSMC migration*.

**Figure 6:**
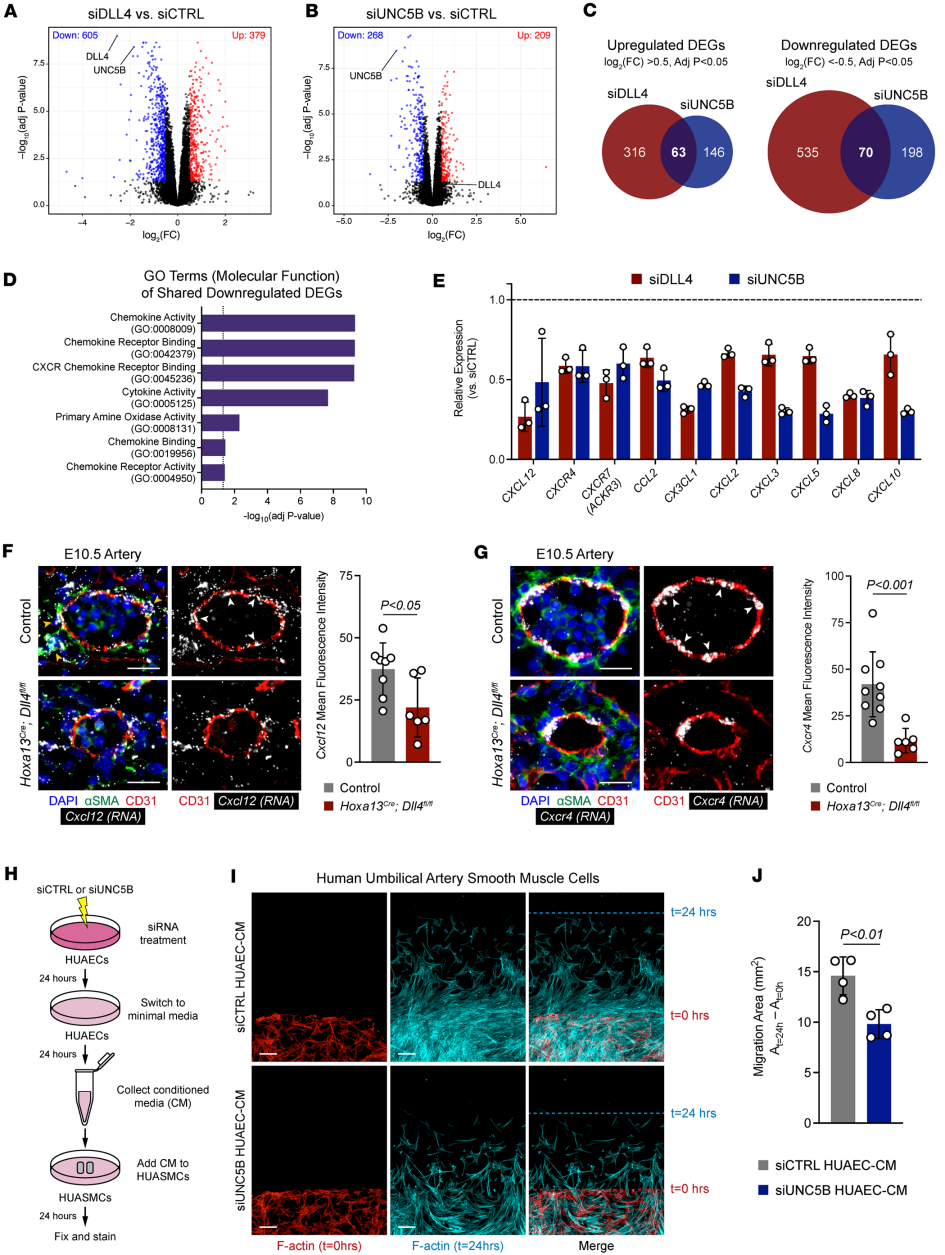
UNC5B controls endothelial chemokine signaling to promote smooth muscle cell migration. (**A** and **B**) Volcano plots of siDLL4- and siUNC5B-treated HUAECs compared with siCTRL restricted to genes with *P*_adj_ < 0.05 and log_2_(Fold Change) < –0.5 and > 0.5. (**C**) Venn diagrams demonstrating number of shared upregulated and downregulated differentially expressed genes (DEGs) between siDLL4- and siUNC5B-treated HUAECs. (**D**) Gene ontology (GO) analysis (molecular function) of the 70 shared downregulated DEGs. Dotted line indicates the threshold for significance. (**E**) Expression of genes contained within chemokine-related GO terms for siDLL4- and siUNC5B-treated HUAECs relative to siCTRL (dotted line) (*n* = 3 independent experiments per condition). All genes are significant with an adjusted *P* < 0.05. (**F** and **G**) Immunofluorescence staining and RNA-FISH of arteries from E10.5 control and *Hoxa13^Cre^;Dll4^fl/fl^* placentas for αSMA (green), CD31 (red), and *Cxcl12* (**F**) or *Cxcr4* (**G**) (gray, RNA) and quantification of mean fluorescence intensity (*n* = 6–9 placentas per genotype, each data point represents the average of at least 3 vessels per placenta). Yellow arrowheads point to *Cxcl12*-expressing vSMCs. White arrowheads point to *Cxcl12*- and *Cxcr4*-expressing ECs. Scale bars: 25 μm. (**H**) Schematic of workflow for conditioned media (CM) experiments (*n* = 4 independent experiments per condition). HUAEC, human umbilical artery endothelial cell; HUASMC, human umbilical artery smooth muscle cell; HUAEC-CM, HUAEC-conditioned media. (**I**) Phalloidin staining and fluorescence of HUASMC migration experiments. Phalloidin was pseudo colored to red for t=0 hrs and cyan for t=24 hrs then overlayed. Dotted red line shows cell edge at t=0 hrs, and dotted cyan line shows cell edge at t=24 hrs. Scale bars: 50 μm. (**J**) Quantification of HUASMC migration cultured in siCTRL HUAEC-CM or siUNC5B HUAEC-CM after 24 hours. Data represent mean ± SD. An unpaired *t* test was performed for statistical analysis.

Analysis of shared differentially expressed genes (DEGs) between siDLL4- and siUNC5B-treated HUAECs using significant genes (*P*__adj__ < 0.05) with a log fold change < –0.5 or > 0.5 were performed to determine the role of *UNC5B* as an effector of *DLL4*. siDLL4-treated cells had 379 significantly upregulated and 605 significantly downregulated genes (Figure 6A), and siUNC5B-treated cells had 209 significantly upregulated and 268 significantly downregulated genes (Figure 6B). Comparing siDLL4 and siUNC5B DEGs, there were 63 shared upregulated DEGs and 70 shared downregulated DEGs (Figure 6C). GO analysis of shared downregulated DEGs found chemokine signaling as a major differentially regulated pathway (Figure 6D). Analysis of these genes found multiple downregulated chemokine-related genes to similar degrees in both siDLL4- and siUNC5B-treated cells (as well as double siDLL4/UNC5B knockdown cells), including *CXCL12* and its receptor *CXCR4* (Figure 6E and Supplemental Figure 5E), which have previously be described to play central roles in arterial development through vSMC recruitment (34–37). While *Cxcl12* expression among ECs is highest in arteries (Supplemental Figure 5F), RNA-FISH staining for *Cxcl12* demonstrated staining in both vSMCs and ECs (Figure 6F) while *Cxcr4* was restricted to only ECs (Figure 6G). Consistent with our in vitro RNA-seq data, placental deletion of *Dll4* decreased arterial endothelial expression of *Cxcl12* and *Cxcr4* (Figure 6, F and G).

Prior work in zebrafish suggests that endothelial *cxcl12* and *cxcr4* function promote vSMC migration and recruitment by controlling secretion of chemoattractants (37). In order to test the effects of *UNC5B* on chemokine signaling in the context of vSMC migration, we designed an in vitro experiment whereby conditioned media from siCTRL and siUNC5B-treated HUAECs was added to human umbilical arterial smooth muscle cells (HUASMCs) and allowed to migrate for 24 hours (Figure 6H). HUASMCs cultured in conditioned media from siUNC5B-treated HUAECs (siUNC5B HUAEC-CM) migrated less than cells cultured in conditioned media from siCTRL-treated HUAECs (siCTRL HUAEC-CM) (Figure 6, I and J, and Supplemental Figure 6). This suggests that *UNC5B* controls secretion of chemoattractants that promote vSMC recruitment. Altogether, these studies and the studies above demonstrate that *UNC5B* is an effector of *DLL4* in simultaneously restricting EC migration via stabilization of cell adhesion and promoting vSMC migration through chemokine signaling.

### *Endothelial CXCR4 signaling promotes vSMC recruitment*.

**Figure 7:**
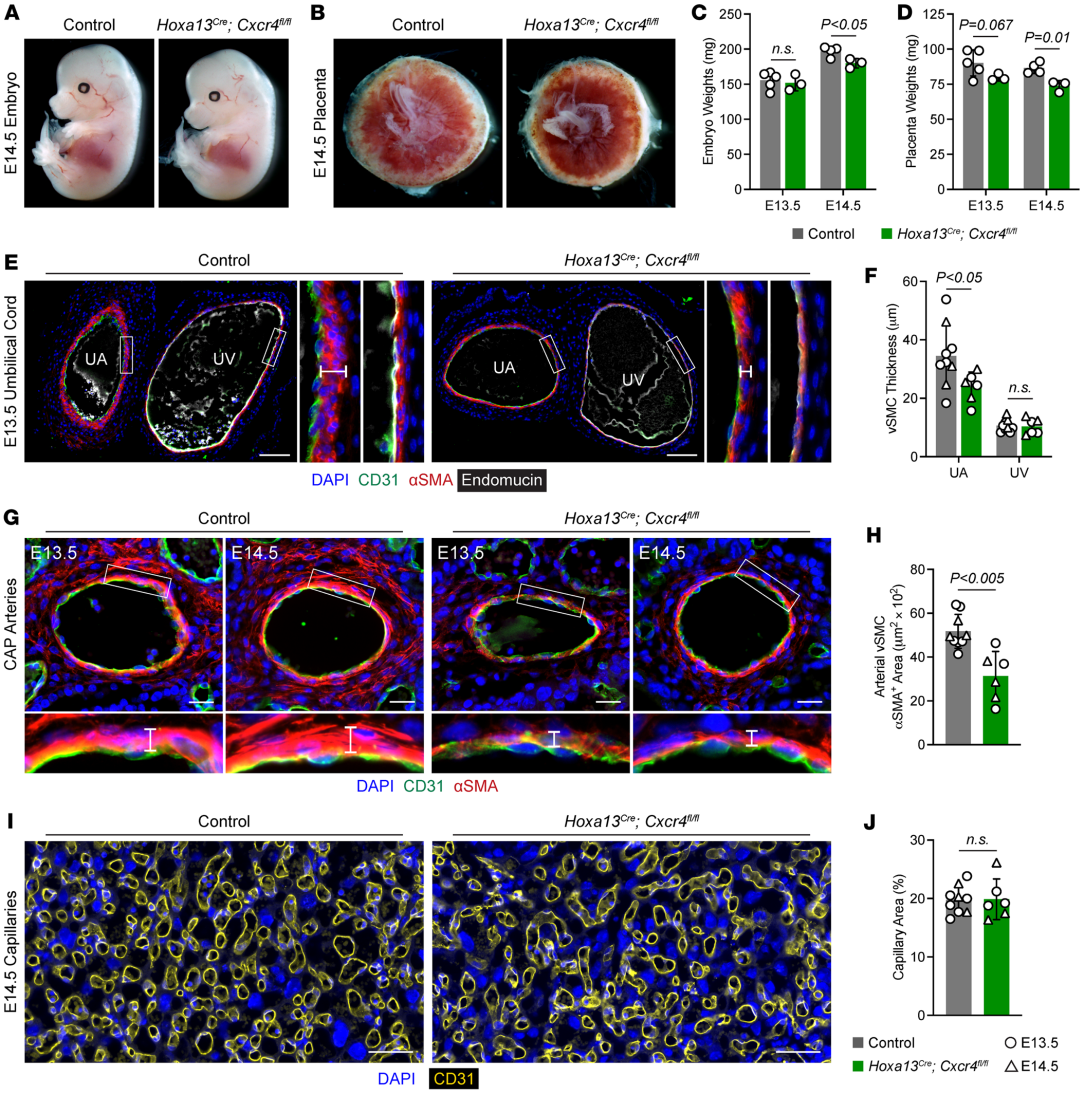
Endothelial *Cxcr4* promotes umbilical and placental artery vSMC recruitment. (**A**–**D**) Gross images of E14.5 control and *Hoxa13^Cre^*;*Cxcr4^fl/fl^* embryos (**A**) and placentas (**B**) and quantification of E13.5 and E14.5 embryo and placenta weights (**C** and **D**) (*n* = 3-5 placentas per genotype). (**E** and **F**) Immunofluorescence staining of E13.5 control and *Hoxa13^Cre^*;*Cxcr4^fl/fl^* umbilical cords for CD31 (green), αSMA (red), and Endomucin (gray) and quantification of perivascular arterial αSMA^+^ vSMC thickness (**F**) (*n* = 6-9 placentas per genotype). UA, umbilical artery; UV, umbilical vein. Scale bars: 100 μm. (**G** and **H**) Immunofluorescence staining of arteries in the chorioallantoic plate in E13.5 and E14.5 control and *Hoxa13^Cre^*;*Cxcr4^fl/fl^* placentas for CD31 (green), αSMA (red), and Endomucin (gray) and quantification of E13.5/14.5 perivascular arterial αSMA^+^ vSMCs (**H**) (*n* = 6–9 placentas per genotype, each data point represents the average of at least 3 vessels per placenta). Scale bars: 25 μm. (**I** and **J**) Immunofluorescence staining of labyrinth capillaries in E13.5 and E14.5 control and *Hoxa13^Cre^*;*Cxcr4^fl/fl^* placentas for CD31 (yellow) and quantification of the capillary area in the labyrinth (**J**) (*n* = 6-9 placentas per genotype). Scale bars: 50 μm. Data represent mean ± SD. An unpaired *t* test was performed for statistical analysis.

Lastly, we examined the role of chemokine signaling in umbilical cord and placental vascular development. Endothelial deletion of *Cxcr4* causes coronary artery defects at E16.5 (34, 36) and reduced vSMC coverage in zebrafish and E12.5 mouse aortas (37). *Hoxa13^Cre^* and *Cxcr4* floxed mice were crossed to generate allantois-specific *Cxcr4*-KO (*Hoxa13^^Cre^^;Cxcr4^fl/fl^*) mice to test the role of chemokine signaling in vivo. *Hoxa13^^Cre^^;Cxcr4^fl/fl^* survived until at least P21 at expected genotypic ratios without grossly obvious defects (Supplemental Table 3). Gross examination of embryos, placentas, and umbilical cords at E13.5 and E14.5 showed no obvious differences (Figure 7, A and B). E13.5 embryo and placenta weights were similar between controls and KOs, but by E14.5, both *Cxcr4*-KO embryos and placentas weighed slightly less (9% and 15% decrease, respectively; Figure 7, C and D). Examination of embryo hearts showed no differences in ventricular thickness at E13.5, but by E14.5, *Hoxa13^^Cre^^;Cxcr4^fl/fl^* embryos had thinner ventricular compact myocardium consistent with growth restriction (Supplemental Figure 7, A–C). Loss of CXCR4 signaling therefore leads to slight embryonic and placental growth restriction by E14.5.

Immunofluorescence staining for Endomucin, CD31, and αSMA on the umbilical cord near placental insertion revealed that *Cxcr4*-KO umbilical cords showed mildly reduced vSMC coverage compared with controls as measured by vSMC thickness (Figure 7, E and F). In contrast, there were no differences in the umbilical vein (Figure 7, E and F). Examination of the CAP arteries similarly showed reduced vSMC coverage in arteries of *Cxcr4* KOs compared with controls; however, there were no differences in labyrinth capillaries coverage (Figure 7, G–J). Together, these data demonstrate that endothelial chemokine signaling in the umbilical artery and placental arteries promotes vSMC recruitment to promote embryonic and placental growth.

## Discussion

The placenta is a complex organ influencing fetal heart and vascular development (22, 38–40). Formation of its vascular network is a multifaceted process during which the allantois fuses with the chorion to form vessels in the umbilical cord and placenta. A lack of genetic tools to target the placenta independent of the embryo has hindered insights into its biology and discovery of new mechanisms of vascularization. Previous work on placental vascular development often involved mesoderm-specific, epiblast-specific, or global KOs in combination with various Cre lines targeting embryonic compartments (endocardial, myocardial, endothelial, hematopoietic) to demonstrate lack of phenotypes (32, 41, 42). In this study, we used a genetic tool previously developed by our lab to demonstrate that the allantois contributes to ECs in the umbilical cord, CAP, and labyrinth in a DLL4-dependent manner.

The allantois has long been known to be a source of progenitor cells for the umbilical cord and placental vasculature. Deletion of *Hoxa13* results in midgestation lethality due to decreased labyrinth ECs and umbilical artery stenosis (26, 43–45). However, whether the umbilical endothelium is derived from *Hoxa13^+^* progenitors in the allantois has yielded inconsistencies (24–26). Two groups have generated Cre lines under the *Hoxa13* promoter for lineage tracing studies. Scotti et al. generated a knockin-knockout *Hoxa13^Cre^* allele replacing the first exon and demonstrate 30%–40% of labyrinth ECs are labeled by fluorescent reporter at E10.5, increasing to 70%–80% at E16.5 (24, 25). However, no umbilical cord ECs are labeled, and they suggest that these ECs are derived from *Hoxa13^–^* progenitors Liang et al. generated a *Hoxa13^IRES-CreER^* allele and show that tamoxifen induction at E8.0 labels 40% of labyrinth ECs at E11.0, but the umbilical cord was not examined (27). In contrast, our previous studies and additional data here show that our *Hoxa13^Cre^* allele labels nearly all labyrinth ECs by E11.5 and at E16.5, including a subset of umbilical cord ECs beginning at E11.5 (23, 46). Our allele may better reflect endogenous gene function due to a T2A self-cleaving peptide in the 3′ UTR that preserves gene function. At least a portion of umbilical cord ECs are derived from *Hoxa13^+^* progenitors, consistent with endogenous expression patterns, with higher contributions closer to the placenta. Differences in findings among studies may reflect different mouse genetic strategies that disrupt endogenous gene function. While it is possible that our *Hoxa13^Cre^* allele does not completely label all cells in the allantois and the contribution is higher, ECs from the embryo proper may also contribute to the umbilical endothelium closer to the embryo.

Deletion of *Dll4* most severely affected large vessels in the umbilical cord and CAP but also affected capillaries. Whether the primary defect is in the umbilical cord or CAP placental arteries is unclear, given higher *Dll4* expression in the umbilical cord but higher allantois contribution to CAP arteries. Most likely a combination of defects in both results in our observed placental phenotypes. *Dll4*-KO placentas also have smaller veins, a finding also observed in global *Dll4* KOs (14, 15), despite venous ECs lacking *Dll4* expression. This is likely secondary to arterial defects through 2 mechanisms: (a) *Dll4* promotes arterial differentiation (13–15), and (b) venous-derived *Dll4^+^* tip cells enable arterial-venous connections (47, 48). As previously reported, we demonstrate that *Dll4* has gene dosage effects in *Hoxa13^^Cre^^*;*Dll4^fl/+^* placentas. However, the effect is milder compared with global heterozygous loss (14, 15), likely due to combinatorial effects of global loss on arteries in the embryo, placenta, and umbilical cord compared with targeted deletion in the placenta.

An important result of careful phenotyping of our mutants is the presence of pericardial edema in *Hoxa13^^Cre^^*;*Dll4^fl/fl^* embryos. Previous studies using global *Dll4* mutants or panendothelial Cre lines (i.e., *Tie2^Cre^*) have observed a similar phenotype and attributed it to cardiovascular collapse in the embryo proper. Our model deletes *Dll4* only in the placental vasculature and demonstrates that the embryonic arterial system is still present and without collapse, albeit dilated with some loss of some smooth muscle. This importantly demonstrates that disrupting vascular development in the placenta alone may account for some of the embryonic phenotypes seen in various global KOs. The *Hoxa13^Cre^* mouse will be an important tool for future efforts in vascular biology and warrants a reevaluation of previous phenotypes that were attributed solely to defects in embryonic cardiovascular development.

We identify *Unc5b* through RNA-seq as an effector of *Dll4*. *Unc5b* belongs to the netrin family of receptors, and previous investigations into the role of *Unc5b* in vascular development is somewhat debated. Lu et al. (33) reported that *Unc5b* is required for embryonic vascular morphogenesis by restricting tip cell identity and suggested this caused cardiovascular failure and embryonic lethality between E10.5 and E12.5 in global KOs due to presence of pericardial edema (33). The placenta was not investigated in this study, and as shown here, placental defects can independently cause pericardial edema. In contrast, Navankasattusas et al. found that panendothelial *Tie2^Cre^*-mediated deletion of *Unc5b* in mice resulted in embryonic lethality at E12.5 with defects only in the placental arterial vasculature, specifically decreased arteriolar smooth muscle (31). Interestingly deletion of *Netrin-1*, a secreted ligand that binds and activates *Unc5b*, results in arterial defects and loss of vSMCs in kidneys (49, 50), which is reminiscent of endothelial deletion of *Unc5b* in the placenta (31). Consistent with our results, a recent study showed that Notch inhibition reduced *Unc5b* expression, and endothelial overexpression of Notch in vivo promoted *Unc5b* expression in arteries and veins (51).

Stabilization of EC junctions has been shown to restrict cell migration (52–54). Notch signaling inhibits cell migration while promoting EC quiescence, lumen formation, and arteriogenesis (19, 20). Similar to our studies, knockdown of *UNC5B* in ECs in vitro decreased junctional VE-cadherin and increased EC migration (51). In our study, we find that *UNC5B* restricts EC migration by stabilizing cell-cell junctions downstream of *DLL4*, suggesting that loss of *Dll4-Unc5b* disrupts large artery formation in part through junctional destabilization. Additionally, *UNC5B* controls chemokine signaling to promote vSMC migration, pointing to potential dual cell autonomous and cell nonautonomous roles for *Unc5b* in coordinating arterial assembly. Whether chemokines secreted by ECs act directly on vSMCs in the placenta remains to be determined. Endothelial-specific deletion of *Cxcr4* or *Cxcl12* causes arterial defects (34–36), and studies in zebrafish suggest that *cxcl12/cxcr4* functions in an endothelial autocrine fashion to promote pdgfb secretion, which then attracts vSMCs (37). Future studies utilizing our *Hoxa13^Cre^* may further clarify the placenta-specific role of *Unc5b* and specific chemokines.

Notably, placental loss of *Dll4* results in early embryonic lethality in contrast to loss of *Cxcr4*, where embryos showed only a mild phenotype with partial loss of vSMC coverage in arteries. This is consistent with previous findings where deletion of *Unc5b*, *Cxcr4*, or *Cxcl12* resulted in embryonic lethality later in gestation (31, 33–36) compared with global *Dll4* KOs. We reconcile these differences by considering that *Dll4*-Notch, but not *Unc5b* or *Cxcr4*, controls arterial identity of ECs as previously demonstrated (6–8, 14–16) and corroborated by our in vitro RNA-seq data. Only a portion of umbilical ECs are derived from the allantois; therefore, loss of *Cxcr4*, which only affects vSMC recruitment and not EC arterial identity, may be more easily compensated by other ECs. Additionally, CXCL12 can signal through both CXCR4 and CXCR7 (55), meaning there may be compensatory signaling. While a *Dll4*-*Unc5b*-chemokine signaling axis has not previously been described, this only encapsulates a single aspect of placental vascular development downstream of *Dll4*. DLL4 has strongest affinity for NOTCH1, which is expressed on both ECs and vSMCs in the placenta, and we demonstrated reduced NOTCH1-ICD in vSMCs suggesting a role for endothelial *Dll4* in promoting vSMC development. However, DLL4 also binds other Notch receptors, such as NOTCH2 (expressed in vSMCs and trophoblasts) and NOTCH3 (expressed in vSMCs) (56–58). *Notch2* and *Notch3* regulate vSMC differentiation, proliferation, and survival (59), and combined *Notch2* and *Notch3* deletion results in embryonic vascular smooth muscle defects and lethality by E11.5 (57). In the placenta, arterial DLL4 may also bind NOTCH2 and NOTCH3 in neighboring vSMCs to regulate their development as a complementary mechanism to UNC5B/chemokine-mediated migration. Ongoing studies in our lab investigating these multifaceted roles of *Dll4*-Notch activation in the placenta through *Hoxa13^Cre^*-mediated deletion of other Notch receptors are ongoing.

A large recent clinical study demonstrated that fetuses with congenital heart disease and placental malperfusion were enriched for de novo variants in Notch signaling, including *NOTCH1*, *NOTCH3*, and *DLL4* (60). However, other smaller studies examining Notch signaling in human placentas from fetuses with intrauterine growth restriction show widely varied results, with some placentas exhibiting reduced Notch signaling (61, 62) and others showing no difference or possibly even increased Notch signaling (63). This is likely in part due to the complexity of Notch signaling in multiple cell types and the fact that fetal growth restriction has multiple etiologies, both embryonic and placental in origin, that could differentially affect Notch signaling. Future studies targeted toward uncovering mechanisms of growth in this unique vascular bed and revisiting previously studied molecular pathways — as well as delineating the placenta-heart axis — will have important implications in understanding placental maladaptation in human disease.

## Methods

### *Sex as a biological variable*.

This study used both male and female animals, with similar findings reported for both. Sex was not considered as a biological variable.

### *Generation of mutant mice*.

*Hoxa13^Cre^* mice have been previously described (23) in which T2A-Cre was inserted into the 3′ UTR region of the endogenous *Hoxa13* locus after Exon 2. *Dll4* floxed mice have been previously described (64) and were generated with LoxP sites flanking exons 1 through 3. Chemokine (C-X-C motif) receptor 4 (*Cxcr4*) floxed mice were purchased from The Jackson Laboratory (strain no. 008767). *Ai14* reporter mice were purchased from The Jackson Laboratory (strain no. 007908). Mice were bred according to standard protocols and maintained on a mixed background. Male *Hoxa13^^Cre^^;Dll4^fl/+^* or *Hoxa13^^Cre^^;Cxcr4^fl/+^* mice were mated to female *Dll4^fl/fl^* or *Cxcr4^fl/fl^* mice. Mating pairs were set up in the afternoon and vaginal plugs checked in the morning. Presence of a vaginal plug indicated E0.5. Cre^^–^^ (*Dll4^^fl/+^^ Dll4^fl/fl^* and *Cxcr4^^fl/+^^ Cxcr4^fl/fl^*) and Cre^^+^^ heterozygous (*Hoxa13^^Cre^^;Dll4^fl/+^* and *Hoxa13^^Cre^^;Cxcr4^fl/+^*) littermates were used as controls.

### *Histology and immunofluorescence analysis*.

Whole mouse embryos or placentas were collected and fixed in 4% paraformaldehyde (PFA) overnight at 4°C prior to dehydration in alcohol and paraffin embedding. Tissue sections underwent to dewaxing and rehydration through xylene and ethanol treatment and were then subjected to H&E staining or processed for immunofluorescence. For immunodetection, 10 mM citrate buffer (pH 6) was used for antigen retrieval, and sections were blocked with 10% donkey serum in 1% BSA prior to primary antibody treatment overnight at 4°C. A list of antibodies can be found in Supplemental Table 4. Fluorescence-conjugated Alexa Fluor secondary antibodies were used (1:500, Invitrogen) according to the primary antibody species and counterstained with DAPI (1:1,000). Sections or tissues were mounted on slides with ProLong Gold Antifade reagent. Signals were detected and images collected using a Zeiss LSM 880 confocal microscope and Zeiss Axio Observer 7 widefield microscope. Images were visualized and quantified using ImageJ/FIJI software (NIH).

### *RNA-FISH*.

For RNAscope, paraffin-embedded sections were prepared as described above and used with the RNAscope Multiplex Fluorescent Reagent Kit v2 (ACDBio 323270) according to the manufacturer’s instructions. Following baking for 1 hour at 60°C and deparaffinization in xylene, slides were incubated with RNAscope Hydrogen Peroxide (for 10 minutes at room temperature), Target Retrieval Reagent (for 15 minutes at 100°C), and Protease Plus (for 30 minutes at 40°C). Sections were hybridized with *Dll4* (ACDBio 319971), *Cxcl12* (ACDBio 422711), *Cxcr4* (ACDBio 425901), 3-plex Positive Control (ACDBio 320881), or 3-plex Negative Control (ACDBio 320871) probes for 2 hours at 40°C and stored in 5× SSC solution overnight. On day 2 of the protocol, sections were incubated with AMP1 (30 minutes at 40°C), AMP2 (30 minutes at 40°C), and AMP3 (15 minutes at 40°C) amplification buffers. After amplification, sections were treated with HRP-C1 solution (15 minutes at 40°C), TSA Vivid Fluorophore 650 (ACDBio 323273 diluted 1:1,500 in TSA Buffer, for 30 minutes at 40°C), and HRP blocker (15 minutes at 40°C). Subsequently, sections were subjected to immunofluorescence with primary and secondary antibodies and imaged as above.

### *Cell culture experiments*.

HUAECs were purchased from PromoCell (C-12202) were cultured in EBM-2 Basal Medium (Lonza CC-3156) supplemented with EGM-2 SingleQuots (Lonza CC-4146). siRNAs against *DLL4* and *UNC5B* were purchased from Thermo Fisher Scientific/Life Technologies (siCTRL 4390843, siDLL4 s29213, siUNC5B s47701) and transfection was done using siPORT Amine (AM4502) according to manufacturer’s instructions. For HUAEC migration experiments, HUAECs were treated with siCTRL, siDLL4, or siUNC5B for 24 hours and then seeded in ibidi 2-well chamber inserts and allowed to attach overnight. Inserts were removed in the morning and cells were allowed to migrate for 24 hours before being fixed in 4% PFA for 30 minutes. For smooth muscle cell conditioned media migration experiments, HUAECs were treated with siCTRL or siUNC5B for 24 hours then switched to EBM-2 Basal Media without supplementation to make conditioned media. HUASMCs were purchased from PromoCell (C-12500) and cultured in Smooth Muscle Cell Growth Medium 2 (Ready to Use) (PromoCell C-22062). HUASMCs were seeded in ibidi 2-well chamber inserts and allowed to attach overnight. Inserts were removed in the morning, conditioned media were added, and cells were allowed to migrate for 24 hours before being fixed in 4% PFA for 30 minutes. For immunocytochemistry, fixed cells were permeabilized in 0.2% TritonX, blocked in 10% donkey serum in 1% BSA, incubated in primary antibody overnight at 4°C, and subjected to secondary antibody treatment prior to fluorescence microscopy. A list of antibodies can be found in Supplemental Table 4.

### *Quantification of immunofluorescence images*.

All images were analyzed using ImageJ/FIJI software (NIH). Percent lineage-positive cells were quantified by manually counting RFP^^+^^ERG^^+^^ double-positive cells and taken as a percentage of total ERG^^+^^ cells. Quantification for EC (Endomucin^^+^^) area and vSMA (αSMA^^+^^) area were done by thresholding images and measuring the area. HUAEC migration area was quantified by measuring the remaining wound area (A__t=24h__), taken as a percentage of the original wound area (A__t=0h__), and subtracting from 100 (100 × [A__t=0h__ – A__t=24h__]/A__t=0h__). HUASMC migration area was quantified by measuring Phalloidin^^+^^ area at t=0 hours (A__t=0h__) and t=24 hours (A__t=24h__) and subtracting t=0 hours from t=24 hours (A__t=24h__ – A__t=0h__). UNC5B fluorescence intensity was quantified by tracing CD31^^+^^ area and measuring UNC5B fluorescence intensity. Quantification for vessel circumference and vessel lumen size were done by manually tracing the vessels.

### *RNA-seq and analysis*.

Total RNA was extracted from cultured cells (*n* = 3 per condition) using Qiagen RNeasy Mini Kit (catalog 74104). Three biological replicates were used per experimental group. Quality control, cDNA synthesis and library preparation was performed by Azenta GeneWiz. Sequencing was performed on an Illumina NovaSeq in 2×150 bp configuration. FastQC (v0.11.9) was used to assess quality of FASTQ files and aligned to homo sapiens GRCh38 using kallisto (v0.48.0). Aligned data were imported into R using Tximport (v1.24.0) and normalized as log__2__ counts per million using edgeR (v3.38.4) (65). Genes containing less than 3 counts per million across all samples were filtered out and all samples were normalized using the calcNormFactors function of edgeR (v3.38.4). Data were transformed using the voom function of the limma package (v3.54.2) (66). Limma was used to determine DEGs between groups and adjusted *P* values were calculated using Benjamini-Hochberg method to account for multiple comparisons. Gene set enrichment analysis was performed using Enrichr (67). Violin plots were generated from single cell RNA-seq datasets generated by Liang et al. (27), which are available in NCBI GEO under accession no. GSE152903. UMAP plots were generated from single nucleus RNA-seq placenta previously generated by Marsh and Blelloch (28).

### *Statistics*.

All data are reported as means with *n* ≥ 3 independent experiments or mice, and data represent mean ± SD. Statistical significance was determined using Welch’s 2-tailed *t* test or 1-way ANOVA with post hoc Tukey HSD for multiple comparisons. Differences between means were considered significant at *P* < 0.05. Significant differences in expected genotypes was calculated using Fisher’s exact test and considered statistically significant at *P* < 0.05.

### *Study approval*.

All procedures were conducted using an approved animal protocol (no. 806811) in accordance with the University of Pennsylvania IACUC.

### *Data availability*.

The RNA-seq data sets have been deposited in the NCBI GEO under accession no. GSE267000. Transgenic mouse lines not available through public repositories are available from MLK under a material transfer agreement with the University of Pennsylvania. Raw data can be found in the Supporting Data Values file.

## Author contributions

DCS, HAA, SN, and MLK conceived and designed the research. DCS, HAA, and SN performed the majority of experiments and acquired data. TCSK performed initial experiments. MC, JY, SG, JB, XC, EN, ATT, PMI, and IM provided experimental support. DCS, HAA, and JAP performed data analysis. DCS and MLK wrote the manuscript. All authors read, edited, and approved the final version of the manuscript.

## Funding support

This work is the result of NIH funding, in whole or in part, and is subject to the NIH Public Access Policy. Through acceptance of this federal funding, the NIH has been given a right to make the work publicly available in PubMed Central.

- NHLBI F30 HL158014 and T32 HL007439 (to DCS).
- NHLBI F30 HL178163 (to SN).
- NHLBI K99 HL175038 (to SG).
- NHLBI F30 HL182205 (to EN).
- NHLBI F30 HL173955 (to JAP).
- Leducq Foundation Placenta in Maternal and Fetal Cardiovascular Health and Disease grant (to MLK).

## Supplementary Material

Supplemental data

Supporting data values

## Figures and Tables

**Table 1:**
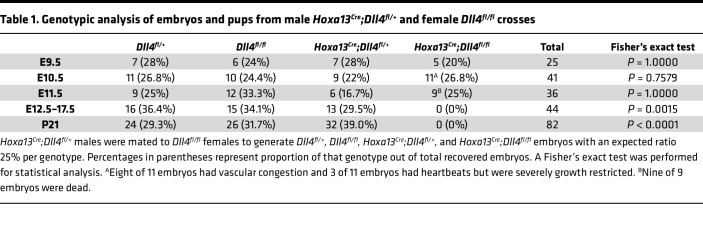
Genotypic analysis of embryos and pups from male *Hoxa13^Cre^;Dll4^fl/+^* and female *Dll4^fl/fl^* crosses

## Version 1 02/17/2026

In-Press Preview

## Version 2 04/08/2026

Electronic publication
