# Supplementary material for: Dll4 assembles the umbilical cord and placental vasculature
Source: JCI Insight. 2026 Feb 17;11(7):e194461. doi: 10.1172/jci.insight.194461 (PMC13134719; doi:10.1172/jci.insight.194461)
Supplement: Supplemental data [file jciinsight-11-194461-s183.pdf]

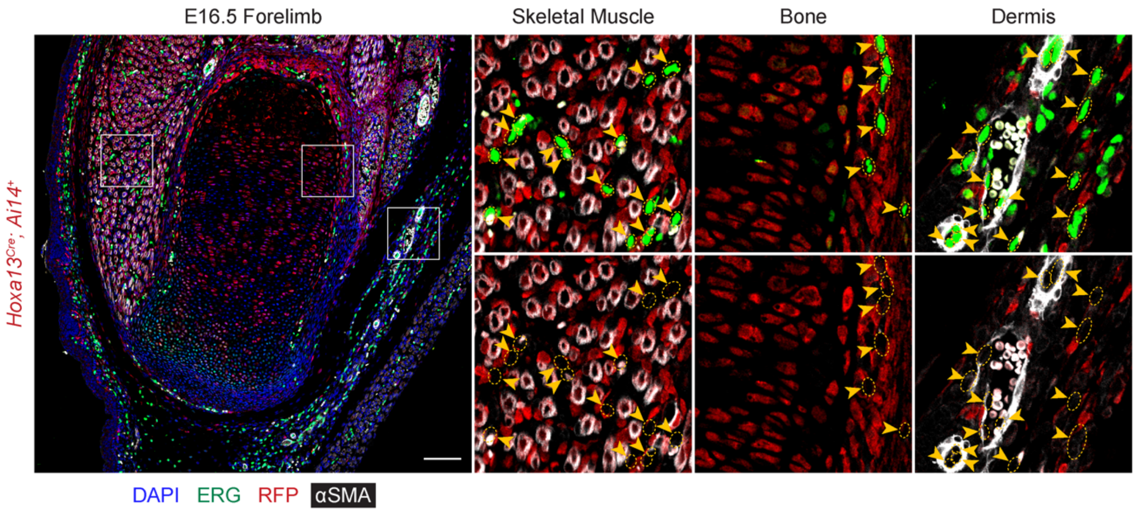

Supplemental Figure 1. *Hoxa13^{Cre}* labels non-endothelial cells in the limbs. Immunofluorescence staining of E16.5 limbs for ERG (green), lineage/RFP (red), and αSMA (grey) in *Hoxa13^{Cre}; Ai14^{+}* embryos. Yellow arrowheads and dotted circles indicate lineage-negative ECs. Scale bars = 100μm.

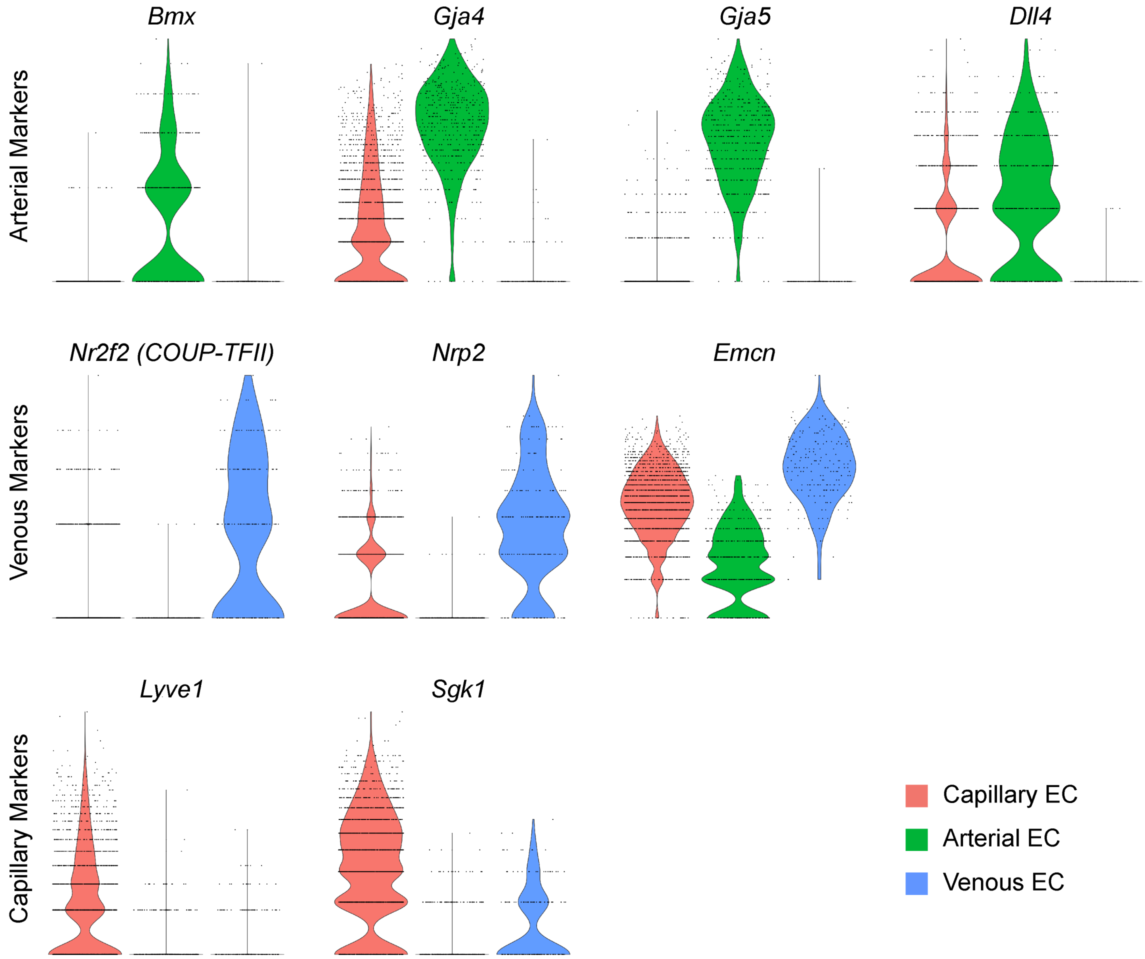

Supplemental Figure 2. Expression of arterial, venous, and capillary markers in placental ECs by single cell RNA-seq. Violin plots of arterial (*Bmx, Gja4, Gja5, Dll4*), venous (*Nr2f2, Nrp2, Emcn*), and capillary (*Lyve1, Sgk1*) genes in placental ECs. Note that expression of *Dll4* is highest in arterial ECs, followed by capillaries, and not expression in veins.

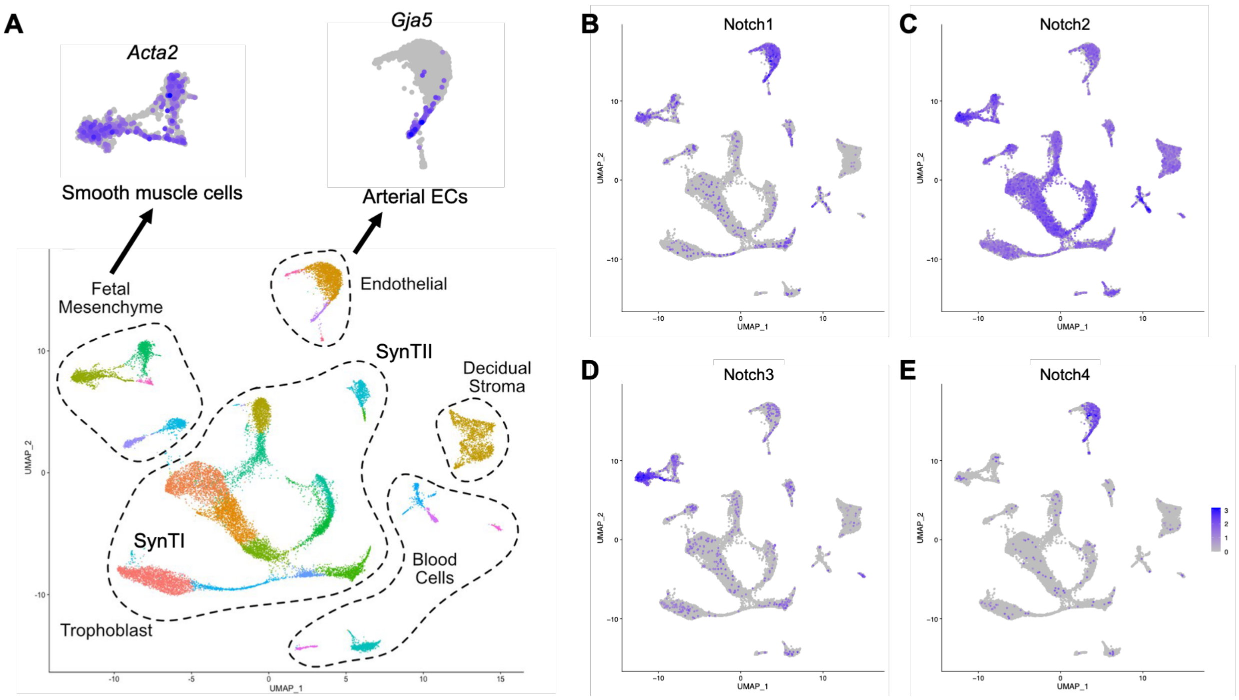

Supplemental Figure 3. Expression profile of Notch receptors in the placenta based on snRNA-seq data. A, UMAP cluster of different cell types in the placenta. *Acta2* is expressed smooth muscle cells in the fetal mesenchyme including arterial vSMCs. *Gja5* is expressed by arterial ECs within the endothelial cluster. **B,** *Notch1* is expressed predominantly in ECs and vSMCs. **C,** *Notch2* is expressed in vSMCs, ECs, trophoblasts, decidual stromal cells, and blood cells. **D,** *Notch3* is expressed predominantly in vSMCs. **E,** *Notch4* is expressed predominantly in ECs. SynT1 = syncytiotrophoblast type 1; SynTII = syncytiotrophoblast type 2

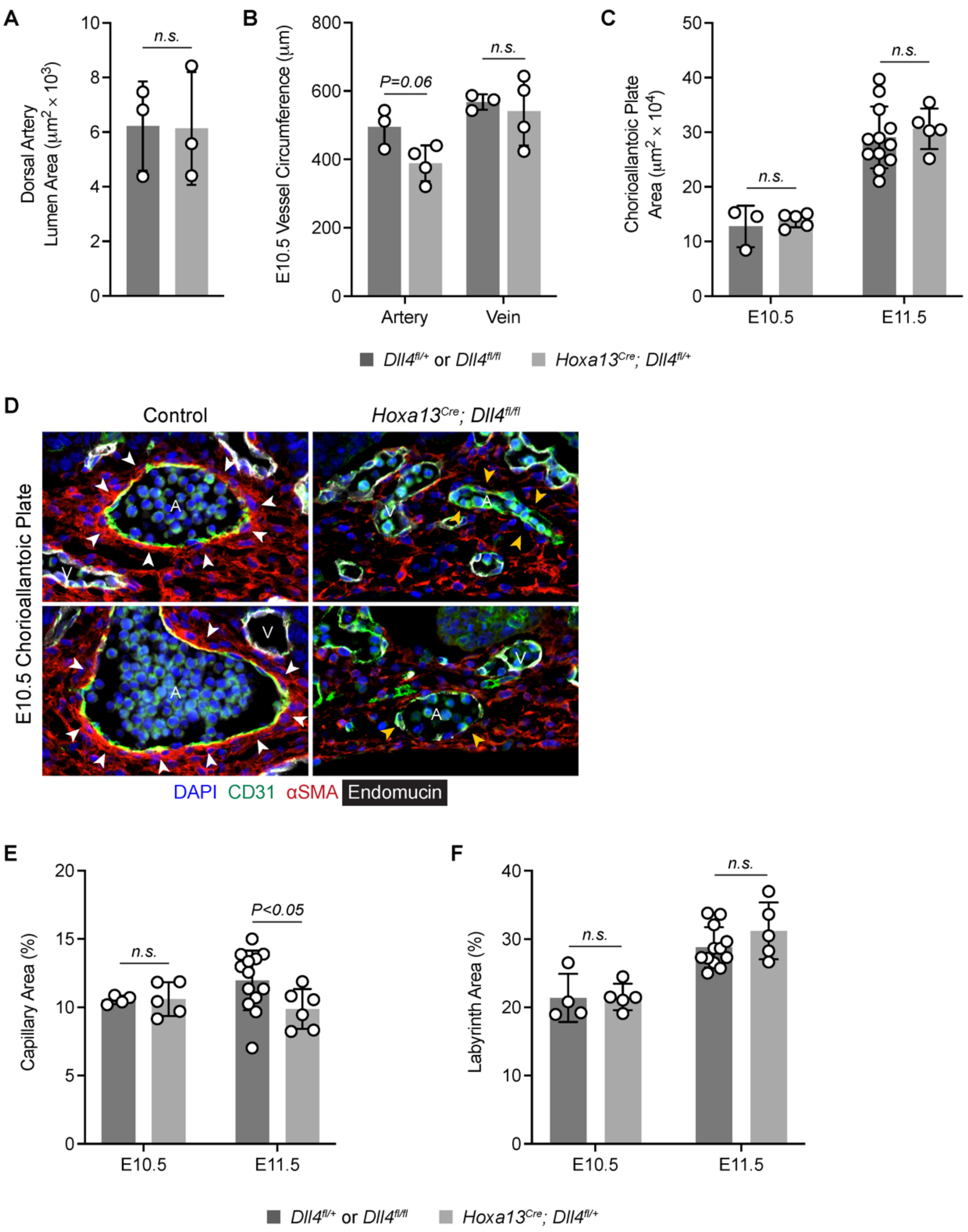

Supplemental Figure 4. Deletion of *Dll4* disrupts chorioallantoic plate growth.
A-C, Comparison of Cre-negative controls (*Dll4*$^{fl/+}$ and *Dll4*$^{fl/fl}$) and Cre-positive heterozygous (*Hoxa13*^{Cre}*; Dll4*$^{fl/+}$) dorsal aortas (**A**), E10.5 placental arteries (**B**), and chorioallantoic plates (**C**) (n=3-12 placentas per genotype). **D,** Additional images of arteries and veins in the chorioallantoic plate. White arrowheads point to abundant αSMA^{+} vSMCs surrounding control arteries whereas yellow arrowheads point to decreased vSMCs in *Hoxa13*^{Cre}*; Dll4*$^{fl/fl}$ arteries. A = artery. V = vein. **E, F,** Comparison of Cre-negative controls (*Dll4*$^{fl/+}$ and *Dll4*$^{fl/fl}$) and Cre-positive heterozygous (*Hoxa13*^{Cre}*; Dll4*$^{fl/+}$) capillary area (**E**) and labyrinth area (**F**) (n=4-13 placentas per genotype). n.s. = not significant. Data represent mean ± SD. An unpaired t-test was performed for statistical analysis.

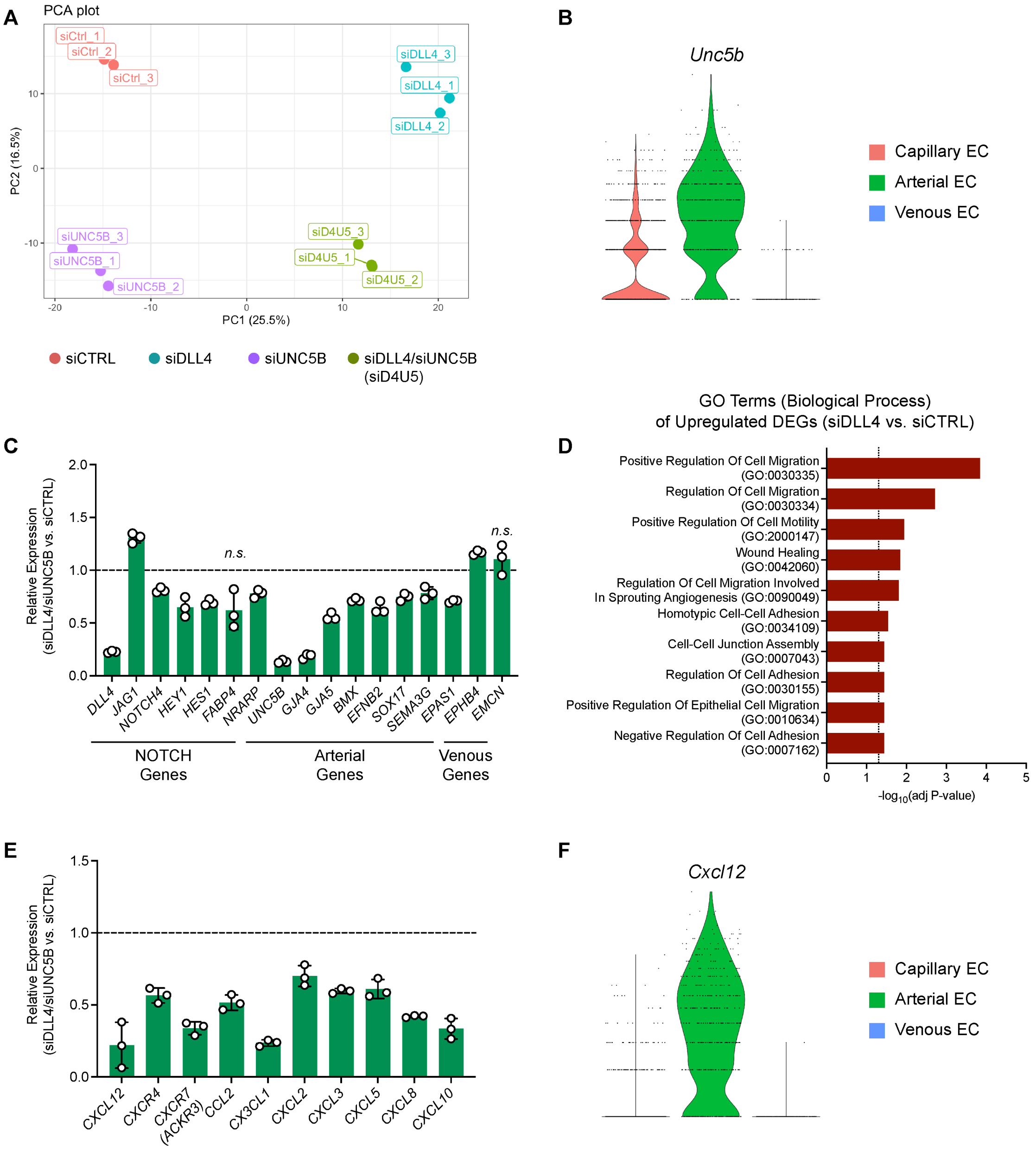

Supplemental Figure 5. Bulk RNA-sequencing analysis of siRNA-treated HUAECs.
A, Principal component analysis of siCTRL, siDLL4, siUNC5B, and siDLL4/siUNC5B-treated HUAECs (n=3 independent experiments per condition). **B,** Violin plot of *Unc5b* expression in placental ECs demonstrating a similar expression profile to *Dll4* with highest expression in arteries followed by capillaries and no expression in veins. **C,** Expression of specific *NOTCH*, arterial, and venous genes in siDLL4/siUNC5B-treated HUAECs relative to siCTRL. All genes are significant with an adjusted P-value <0.05 unless denoted with n.s. (not significant). **D,** Gene ontology (GO) analysis (biological process) of the 1608 upregulated genes in siDLL4-treated HUAECs related to cell migration and cell adhesion. Dotted line indicates the threshold for significance. **E,** Expression of genes contained within chemokine-related GO terms for siDLL4/siUNC5B-treated HUAECs relative to siCTRL (dotted line). All genes are significant with an adjusted P-value <0.05. **F,** Violin plot of *Cxcl12* expression in placental ECs demonstrating expression only in arteries.

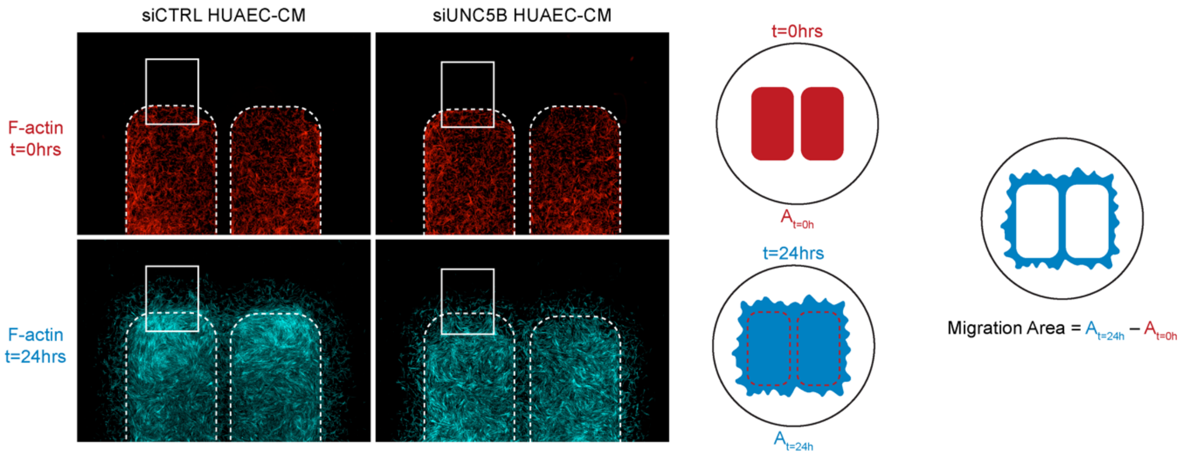

Supplemental Figure 6. Schematic showing calculation of migration area in HUASMC conditioned media experiments. Phalloidin staining and fluorescence of HUASMC migration experiments. Phalloidin was pseudo colored to red for t=0hrs and cyan for t=24hrs. Dotted white lines outline starting cell edge at t=0hrs. The white box correlates to magnified images shown in **Figure 6I**. Schematic to visualize starting cell area at t=0hrs and ending cell area at t=24hrs and how migration area was calculated. HUAEC = human umbilical artery endothelial cell. HUASMC = human umbilical artery smooth muscle cell. HUAEC-CM = HUAEC-conditioned media.

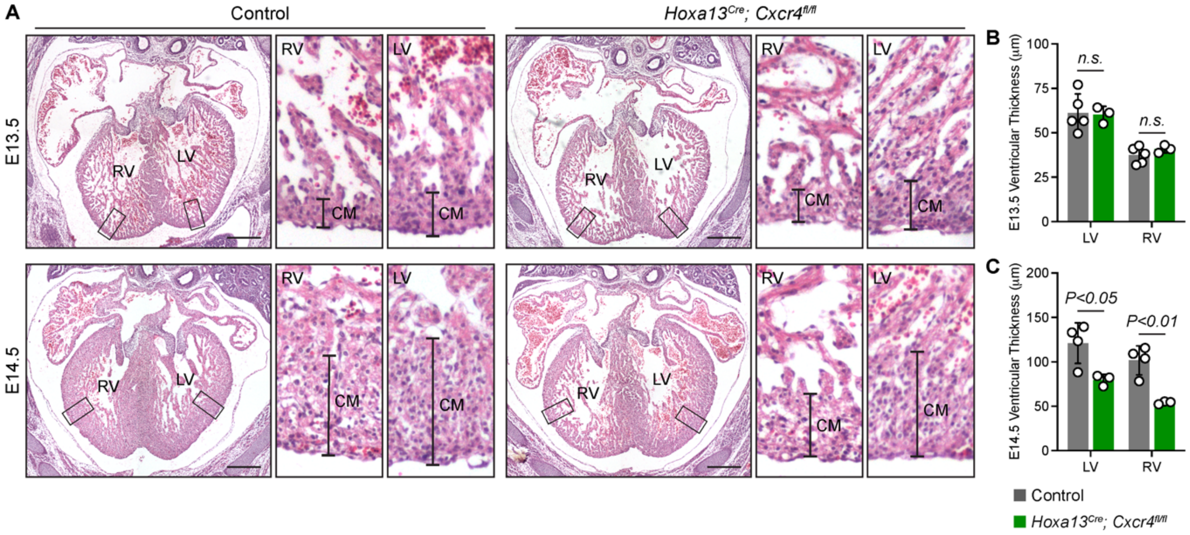

Supplemental Figure 7. Placental *Cxcr4* deletion causes myocardial thinning at E14.5. **A,** H&E images of E13.5 and E14.5 control and *Hoxa13*^{Cre}*; Cxcr4*$^{fl/fl}$ hearts. RV = right ventricle, LV = left ventricle, CM = compact myocardium. Scale bars = 500μm. **B, C,** Quantification of E13.5 (**B**) and E14.5 (**C**) control and *Hoxa13*^{Cre}*; Cxcr4*$^{fl/fl}$ compact myocardial thickness (n=3-5 embryos per genotype). n.s. = not significant. Data represent mean ± SD. An unpaired t-test was performed for statistical analysis.

**Regulation Of Cell Migration (GO:0030334)**<br>*ITGB1, FLT1, SERPINE2, FAM107A, PTPRK, CLDN1, DOCK10, CDH5, DPYSL3, EPHB2, SEMA6C, EPHA4, ANXA1, LIMCH1, F2R, ADAM10, PRKCA, KIF2A, ADAM9, CDH13, ITGA6, ENG, BEX4, KANK1, ROCK1, SRC, SEMA3G, NEDD9, PRKX, SEMABF, EGFR, PODXL, PDGFD, MGAT5, PLXNA2, PLXNA4, SEMA4A, TCAF2, TCAF1, JAG1, YTHDF3, FZD4, SEMA4B, RCC2, SEMA4F, PODN, PTK2, SMAD7, STK24, RECK*
**Positive Regulation Of Cell Migration (GO:0030335)**<br>*ITGB1, BMPR2, FLT1, SRC, FAM107A, SEMA3G, NEDD9, SEMA3F, ACVR1B, CLDN1, EGFR, CDH5, CAPN7, PODXL, PDGFD, MGAT5, EPHB2, EPHA4, SEMAGC, TCAF2, SEMA4A, FZD4, PRKCE, SEMA4B, F2R, DABZIP, ADAM10, PRKCA, SEMA4F, PTK2, RAPGEF2, ADAM9, CDH13, ITGA6, DOCK1, DDRZ*
**Positive Regulation Of Cell Projection Organization (GO:0031346)**<br>*LYN, NCOA2, NDRG4, NLGN1, TENM3, BDNF, FZD4, KIDINS220, DAB2IP, P3H1, NEDD9, PTK7, DPYSL3, PLXNA2, RAPGEF1, RAPGEF2, ROR1, ITGA6, OBSL1, MARK2 PLXNA4, DDR2*
**Regulation Of Wound Healing (GO:0061041)**<br>*ITGB1, ARFGEF1, KANK1, ANXA1, PRKCE, FZR, NRG1, CLDN1, PTK2, MMRNT, CD109, EPHB2, CRK*
**Regulation Of Cell Adhesion (GO:0030155)**<br>*LYN, EPHA4, ZFHX3, ROCK1, SC, ADAM10, PRKCA, NRG1, PRKX, GPR4, PTPN11, EFNA5, CELSR2, PTK2, PODXL, RASA1, PLXNA2, CDH13, TGFBI, EPHB2, PLXNAA*
**Negative Regulation Of Cell Adhesion (GO:0007162)**<br>*EPHA4, JAG1, FZD4, ADAM10, NRG1, PTK2, PODXL, RASA1, PLXNA2, CDH13, SPOCK1, TGFBI, EPHB2, PLXNA4*
**Positive Regulation Of Cell Motility (GO:2000147)**<br>*ITGB1, FLT1, FAM107A, SEMA3G, NEDD9, SEMA3F, CLDN1, EGFR, CDH5, PODXL, PDGFD, MGAT5, EPHB2, EPHA4, SEMA6C, TCAF2, SEMA4A, FZD4, SEMA4B, F2R, ADAM10, PRKCA, SEMA4F, PTK2, ADAM9, CDH13, ITGA6*
**Positive Regulation Of Epithelial Cell Migration (GO:0010634)**<br>*SCARB1, BMPR2, SPARC, SC, IQSEC1, ITGB3, PDCD6, PRKCE, PRKCA, PTK2, CAPN7, ADAM9, CAL, TEK, PLPP3, DOCK1*
**Regulation Of Cell-Cell Adhesion (GO:0022407)**<br>*TENM3, JAG1, SRC, PODXL, EFNA5, PLPP3, CELSR2, PTK2, PTPRG, SMAD7*

Supplemental Table 1. List of upregulated genes for cell migration and cell adhesion-related GO terms (biological process) for siUNC5B-treated HUAECs

**Positive Regulation Of Cell Migration (GO:0030335)**<br>*ITGB1, BMPR2, CIB1, CLDN1, CRKL, CDH5, PLAU, RACK1, EPHB2, SUN2, PRKCE, ADAM10, PRKCA, ACTN4, SHTN1, MYADM, ADAM9, CDH13, ITGA6, PFN1, DDR2, SEMA3C, SEMABA, PDGFA, SEMA3F, ACVR1B, RTN4, PDCD10, PODXL, MGAT5, ZNF703, S100A11, TNFSF18, TCAF2, XBP1, HSPA5, SMURF2, FZD4, SEMA4B, SEMA4C, FN1, LAMB1, DAB2, MYO1C, AGO2, TRIP6, FERMT2, BCAR1, FERMT3*
**Regulation Of Cell Migration (GO:0030334)**<br>*ITGB1, CITED2, CIB1, CLDN1, FGF2, ARHGAP4, AMOT, CDH5, PLAU, DPYSL3, RACK1, DAG1, EPHB2, SUN2, ADAM10, PRKCA, ACTN4, FRMD5, KIF2A, ADAM15, MMRN2, MYADM, ADAM9, CDH13, ITGA6, SGK1, VCL, SEMA3C, PDGFA, NEXN PRKX, SEMA3F, FOX03, CORO1C, PDCD10, PODXL, MGAT5, ZNF703, LMNA, PLXNA2, FLNA, PLXNA1, PLXNA4, ARB, TCAF2, XBP1, JAG1, HSPA5, FZD4, SEMA4B, SEMA4C, LAMB1, SULF1, DAB, MYO1C, STK26, TRIP6, EPPK1, NF2, RECK, FERMT2, BCAR1, FERMT3*
**Positive Regulation Of Cell Motility (GO:2000147)**<br>*ITGB1, SEMA3C, CCDC25, PDGFA, CIB1, SEMA3F, CLDN1, CDH5, PDCD10, PLAU, PODXL, MGAT5, ZNF703, RACK1, EPHB2, TCAF2, SUN2, XBP1, HSPA5, FZD4, SEMA4B, SEMA4C, ADAM10, PRKCA, LAMB1, ACTN4, DAB2, MYO1C, TRIP6, MYADM, ADAM9, CDH13, ITGA6, FERMT2, BCAR1, FERMT3*
**Wound Healing (GO:0042060)**<br>*SCARB1, ITGB3, MYOF, PDGFA, NRG1, PRCP, CLDN1, FGF2, CORO1B, SYT11, ERBB2, CHMP2B, ANXA6, MYH9, HMOX1, EPPK1, PLPP3, PLEC*
**Regulation Of Cell Migration Involved In Sprouting Angiogenesis (GO:0090049)**<br>*STARD13, PDCD10, MMRN2, RHOJ, HMOX1, CIB1, MEOX2, PIK3C2A, PTGS2, FGF2*
**Homotypic Cell-Cell Adhesion (GO:0034109)**<br>*CSRP1, UBASH3B, ITGB3, ACTN1, ILK, FLNA, MYH9, PLPP3, TLN1, CD99, VCL, FERMT3*
**Cell-Cell Junction Assembly (GO:0007043)**<br>*CNTNAP1, WDR1, JUP, CTNND1, PRKCA, CLDN11, CDH5, CDH2, EPB41L3, ZNF703, FSCN1, CDH13, CD9, TLN1, VCL*
**Regulation Of Cell Adhesion (GO:0030155)**<br>*TNFSF18, ZFHX3, DUSP1, PLAUR, ADAM10, PRKCA, NRG1, PRKX, LAMB1, FRMD5, PLAU, PODXL, FES, ERBB2, MYADM, PLXNAZ, LPXN, PLXNA1, CDH13, RSU1, TGFBI, EPHB2, TNPO1, PLXNA4*
**Positive Regulation Of Epithelial Cell Migration (GO:0010634)**<br>*GPI, SCARB1, BMPR2, ANXA3, ITGB3, PDCD6, PRKCE, PRKCA, FGF2, RTN4, BMP4, ADAM9, TEK, PFN1, PLPP3, MAPRE2, BCAR1, ARF6*
**Negative Regulation Of Cell Adhesion (GO:0007162)**<br>*JAG1, DUSP1, FZD4, ADAM10, NRG1, PODXL, PLXNAZ, CDH13, LPXN, PLXNA1, SPOCK1, NF2, TGFBI, EPHB2, PLXNA4*

Supplemental Table 2. List of upregulated genes for cell migration and cell adhesion-related GO terms (biological process) for siDLL4-treated HUAECs

	Cxcr4$^{fl/+}$	***Cxcr4$^{fl/fl}$***	***Hoxa13^{Cre}; Cxcr4$^{fl/+}$***	***Hoxa13^{Cre}; Cxcr4$^{fl/fl}$***	**Total**	**Fisher Exact Test**
**P21**	3 (25%)	3 (25%)	2 (16.7%)	4 (33.3%)	12	P=1.000

Supplemental Table 3. Genotypic analysis of pups from ♂ *Hoxa13^{Cre}; Cxcr4$^{fl/+}$* × ♀ *Cxcr4$^{fl/fl}$* crosses. *Hoxa13^{Cre}; Cxcr4$^{fl/+}$* males were mated to *Cxcr4$^{fl/fl}$* females to generate *Cxcr4$^{fl/+}$*, *Cxcr4$^{fl/fl}$*, *Hoxa13^{Cre}; Cxcr4$^{fl/+}$*, and *Hoxa13^{Cre}; Cxcr4$^{fl/fl}$* embryos with an expected ratio 25% per genotype. Percentages in parentheses represent proportion of that genotype out of total recovered mice. A Fisher Exact test was performed for statistical analysis.

**Antibody**	**Species**	**Product Number**	**Application and Dilution**
αSMA-FITC conjugated	Mouse	Sigma C6198	IHC-IF (1:300)
αSMA-Cy3 conjugated	Mouse	Sigma F3777	IHC-IF (1:300)
ERG	Rabbit	Abcam ab92513	IHC-IF (1:200)
RFP	Goat	SicGen AB1140	IHC-IF (1:200)
Cytokeratin 8	Rabbit	Abcam ab53280	IHC-IF (1:300)
TIE2	Goat	R&D AF762	IHC-IF (1:100)
CD31	Goat	R&D AF3628	IHC-IF (1:200)
Ki67	Rabbit	Abcam ab16667	IHC-IF (1:100)
Endomucin	Rat	Abcam ab106100	IHC-IF (1:200)
Endomucin	Goat	R&D AF4666	IHC-IF (1:300)
MCT1	Chicken	Millipore AB1286-I	IHC-IF (1:100)
MCT4	Rabbit	Millipore AB3314P	IHC-IF (1:100)
NOTCH1 ICD	Rat	DSHB bTAN 20	IHC-IF (1:100)
UNC5B	Goat	R&D AF1006	IHC-IF (1:100)
VE-cadherin	Goat	R&D AF938	ICC-IF (1:300)
β-catenin	Rabbit	Abcam ab32572	ICC-IF (1:300)

Supplemental Table 4. List of antibodies, species, product number, and dilutions for specific applications.
